# The Effect of an Orthopaedic Surgeon's Attire on Patient Perceptions of Surgeon Traits and Identity: A Cross-Sectional Survey

**DOI:** 10.5435/JAAOSGlobal-D-20-00097

**Published:** 2020-08-03

**Authors:** Stephanie D. Goldstein, Emma L. Klosterman, Scott J. Hetzel, Brian F. Grogan, Kathryn L. Williams, Ronald Guiao, Andrea M. Spiker

**Affiliations:** From the Department of Orthopedics and Rehabilitation (Dr. Goldstein, Dr. Klosterman, Dr. Grogan, Dr. Williams, Dr. Guiao, Dr. Spiker), University of Wisconsin-Madison, Madison, WI, and the Department of Biostatistics and Medical Informatics (Mr. Hetzel), University of Wisconsin-Madison, Madison, WI.

## Abstract

In orthopaedic surgery, limited research is available addressing how attire, including white coats (WC) and feminine attire such as skirts, affects patient perceptions. It is unknown how surgeon appearance is associated with identification as a surgeon and perception of clinical skills, nor whether this differs between male and female surgeons.

**Methods:**

An image-based survey was offered to all adult patients in four orthopaedic surgeons' sport medicine or foot and ankle clinics. Respondents viewed 10 photos of male and female models in varying attire and identified that individual's most likely role on the healthcare team. Then, in 10 photos pairs, respondents selected which surgeon showed more competence, ability to excel in performing the physical components of surgery, likelihood to provide a good surgical outcome, and trustworthiness.

**Results:**

Two hundred thirty-eight patients participated in the survey. Men were identified as surgeons significantly more frequently than women in similar clothing (*P* < 0.05) for all attire except a business attire without a WC (men: 18.2% vs women: 11.2%; *P* = 0.252). Patients ranked physicians wearing a WC with any attire as more competent and more likely to give a good surgical outcome than those without (all *P* < 0.005). Patients found women in feminine attire significantly less likely to excel in performing the physical parts of surgery than women in scrubs (*P* = 0.001).

**Discussion:**

Women surgeons who wear feminine business attire instead of scrubs may be perceived less able to perform the physical work of operating, but are otherwise rated comparably with their peers, both male and female. Surgeons wearing WC are generally perceived more favorably than those without WC, an effect that is magnified for perceptions of competence for female surgeons. Men are more readily identified as surgeons than women when wearing a similar attire, except for a business attire without WC. There are continuing differences in how patients perceive male and female orthopaedic surgeons based on their attire.

Physician appearance and attire has been the subject of numerous publications over the past three decades. As an easily modifiable factor that may influence patient perceptions, satisfaction, and even outcomes, there has been understandable interest in defining the ideal attire that correlates most closely with desirable professional traits. A recent multicenter study including over 4000 patients receiving nonsurgical inpatient or outpatient care found that formal attire with a white coat (WC) was the most highly rated attire and that one-third of patients agreed that their doctor's appearance influenced their satisfaction with care.^1^ However, the physician traits that are most desirable and salient for patients may vary among medical specialties. Orthopaedic surgery is unique in that it involves manual tasks in the operating room that patients may associate with a need for physical strength, which is more commonly associated with men than women. Although the number of women in orthopaedic training and practice is slowly increasing, orthopaedic surgery remains a male-dominated specialty. As of 2016, only 4% of the members of the American Academy of Orthopaedic Surgeons (AAOS) and 13% of orthopaedic surgery residents were women.^2^ A number of published studies look specifically at the role of surgeon appearance in orthopaedic surgery, many of which feature male and female surgeons in varying attire.^3,4,5,6^ No study to date has looked specifically at the impact of feminine attire, such as skirts or dresses, on patient perceptions of orthopaedic surgical skill, including the ability to excel in performing the physical components of surgery.

The WC, historically worn primarily by physicians, has since become a marker of the healthcare profession more broadly. Orthopaedic surgeons rely heavily on physician extenders, including physician assistants (PAs) and nurse practitioners, to improve many aspects of their practices.^7,8^ Many nonphysicians involved in orthopaedic care may don a WC during their clinical duties. In the authors' experiences, youthful-appearing surgeons and female surgeons have experienced misidentification as a nonphysician member of the healthcare team, such as a nurse or PA. The extent to which a WC helps distinguish the surgeon from other members of the healthcare team and how this differs between male and female physicians remains unknown.

We sought to answer the following questions:

(1) Does feminine business attire affect patient perceptions of four realms of surgical and clinical skills (competence, ability to provide a good surgical outcome, ability to excel in the physical parts of surgery, and trustworthiness) or identification as a surgeon?
(2) Does wearing a WC affect patient perceptions of four realms of surgical and clinical skills or identification as a surgeon?
(3) How readily do patients identify women as surgeons as compared to men when an identical attire is worn?
(4) Are female surgeons in any attire perceived differently than male surgeons?

Based on existing literature of bias in the medical profession,^9,10^ we hypothesized that female models wearing feminine attire would be rated less favorably than men for traits referencing surgical skill and strength, that patients would rate models wearing WCs as more favorable and more likely to be surgeons, and that women will be identified as surgeons less frequently than males.

## Methods

This study was approved by our institutional review board. Four fellowship-trained orthopaedic surgeons in an academic practice recruited patients from their outpatient clinics. Two of these orthopaedic surgeons subspecialized in sport medicine and two in foot and ankle, with one male and female surgeon in each subspecialty. All patients older than the age of 18 presenting to an eligible outpatient orthopaedic clinic were invited to participate in the study before being seen by a physician extender or the attending surgeon. Patients were informed that their participation was voluntary and that their responses were anonymous. They were given the option to take the survey in paper form, in electronic form on a provided tablet, or via a QR code on their smartphone. Questions could be skipped in all versions.

During the study period, participating surgeons wore consistent attire to each clinic. The type of attire was based on personal preference. Female surgeon XX wore feminine a business attire (skirts or dresses) without a WC, female surgeon XX wore business clothing with a WC, male surgeon XX wore a business attire with a WC, and male surgeon XX wore either a business attire without a WC or scrubs with a WC.

**Figure 1:**
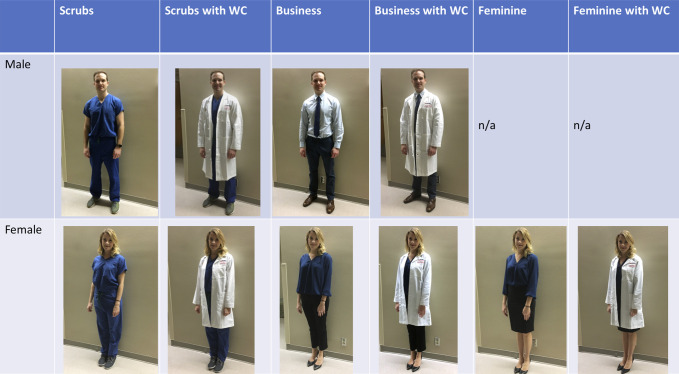
Photographs of male and female models used for identification portion of survey.

**Figure 2:**
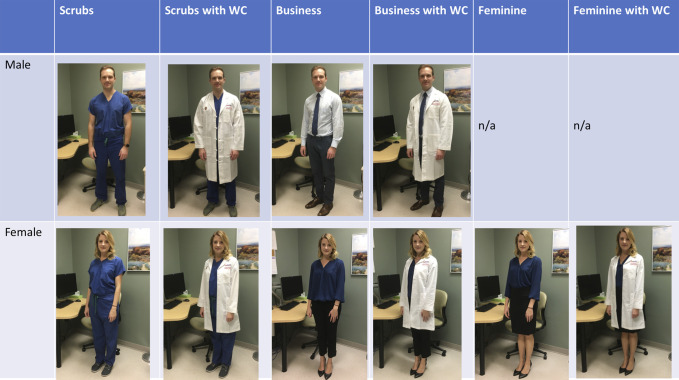
Photographs of male and female models used for comparison portion of survey.

An image-based survey was created comprising three parts (see supplemental materials, http://links.lww.com/JG9/A81). The first collected demographic information, including sex, age, race, education level, home setting (urban, suburban, or rural), and type of visit (new, returning, postoperative, or emergency department follow-up). The second “identification” section presented 10 photos of our male or female model in varying attire and asked respondents to identify their most likely role on the healthcare team (surgeon, PA, nurse, medical assistant, hospital administrator, or other). Descriptions of each role were provided as needed. Photos used in this section were taken in a hallway to make them applicable to either an inpatient or outpatient setting (Figure 1). The third “comparison” section presented one of 10 pairs of photos and asked respondents to select which surgeon fit more closely with one of four traits: competence, ability to excel in performing the physical parts of surgery, likely to provide a good surgical outcome, and trustworthiness. An option of “they seem the same” was also provided. The 10 photograph pairings were chosen to answer the questions outlined in the introduction section (Figure 2). The order of pictures within a question and a given section were randomized.

An internal medicine intern and an orthopaedic surgery intern who had not yet worked in the participating orthopaedic subspecialty clinics volunteered as models and provided consent for his/her image to be used in publication. The male model images included a business attire (blue shirt, tie, and dark pants) and scrubs, each with and without a WC. The female model images included a business attire (blue blouse and dark pants), feminine business attire (same blue blouse amd dark skirt), and scrubs, each with and without a WC.

Statistical analyses were based on the following hypothesis questions: (1) What types of attire and model sex combinations were most likely to be classified as a surgeon? (2) Which picture in the direct comparisons did patients say was most consistent with the listed trait? (3) Did any patient characteristics influence the outcomes of questions 1 and 2?

We calculated the percentage rate of surgeon classification for each of the 10 identification photos. We compared the percentages in all two-way comparisons via Fisher exact test with Holm adjustment for 45 multiple comparisons. To determine which picture was selected most frequently in the comparison section, we removed “they seem the same” responses, calculated the percentage for one of the pictures in the dyad, and tested the percentage by a two-sided test of a single proportion versus a null hypothesis of 50%, which is the expected rate because of random chance. This was repeated for each of the four subquestions. *P* values from the 10 tests within each subquestion were Holm adjusted to account for multiple testing. Comparisons based on patient characteristics were done via Fisher exact tests and also used Holm adjustments for multiple comparisons. An adjusted 5% significance level was used for all tests. Analysis was performed in R for statistical computing version 3.5.

## Results

From August to October 2019, 238 patients participated in the survey, representing 32% of the eligible clinic population. Participant demographics and clinic information are in Table 1. Nearly all (95.1%) of the respondents reported that they did not recognize the models in the survey photos. Most patients completed the surveys on a tablet, whereas 6.7% elected to complete the survey on paper. The most common reason for the appointment was a postoperative visit (43.6%); male and female orthopaedic surgeons were nearly equally likely to have performed a surgery on their patients (48% versus 52% of postoperative patients, respectively). More than half (57.5%) of the respondents reported that their primary care physician was the same sex as themselves, whereas 47.5% of respondents were the same sex as their orthopaedic surgeon.

**Table 1:** Characteristics of Study Respondents and Clinic Attendings

Characteristics	Total Survey Responses = 238
Patient age	n = 238
18-25	24 (10.1%)
26-34	29 (12.2%)
35-49	59 (24.8%)
50-65	88 (37.0%)
66-80	36 (15.1%)
81+	2 (0.8%)
Patient sex	n = 217
Male	98 (45.4%)
Female	118 (54.4%)
Highest education achieved	n = 235
Before high school	2 (0.9%)
High school graduate	40 (17.4%)
Some college	87 (37.8%)
Bachelor's degree	64 (27.8%)
Master's degree	28 (12.2%)
Doctorate degree	9 (3.9%)
Race	n = 242^a^
Caucasian	223 (92.2%)
Black or African American	4 (1.7%)
Hispanic	6 (2.5%)
American Indian or Alaska Native	4 (1.7%)
Asian	2 (0.8%)
Native Hawaiian or Pacific Islander	1 (0.4%)
Other	1 (0.4%)
Living area	n = 234
Rural	76 (32.5%)
Suburban	91 (38.9%)
Urban	67 (28.6%)
Appointment type	n = 234
New patient	58 (24.8%)
Postoperative visit	102 (43.6%)
Returning patient	68 (29.1%)
Emergency department follow-up	0 (0%)
Other	6 (2.6%)
Attending specialty	n = 229
Sport medicine	168 (73.4%)
Foot & ankle	61 (27.6%)
Attending sex	n = 229
Male	110 (48.0%)
Female	119 (52.0%)
Patient PCP sex	n = 206
Male	86 (41.8%)
Female	104 (50.5%)
Do not have/prefer not to say	16 (7.7%)
Sex-matched PCP^b^	119 (57.5%)

PCP = primary care physician

a Each respondent was allowed to select more than one response.

b Indicates patients whose sex matches that of their reported PCP.

Respondent age, sex, education level, and the subspecialty and sex of the surgeon they saw in clinic did not influence responses to the comparison questions. Respondents younger than the age of 50 were more likely to identify a woman in a business attire and WC as a surgeon than those older than 50 (70.4% vs 50.4%; *P* = 0.032), who associated this attire with nonsurgeons, primarily PAs. Respondents younger than 50 years old identified women in scrubs only as surgeons more frequently than did their older than 50 counterparts, but this did not reach statistical significance after multiple comparison adjustment (28.7% vs 13.9%; *P* = 0.087). Those who were college graduates identified women in a business attire without WC as surgeons more frequently than noncollege graduates (18.7% vs 4.8%; *P* = 0.016). For the remainder of the identification questions, no differences between age groups were found for other types of attire, nor for the other above-listed demographic criteria.

**Figure 3:**
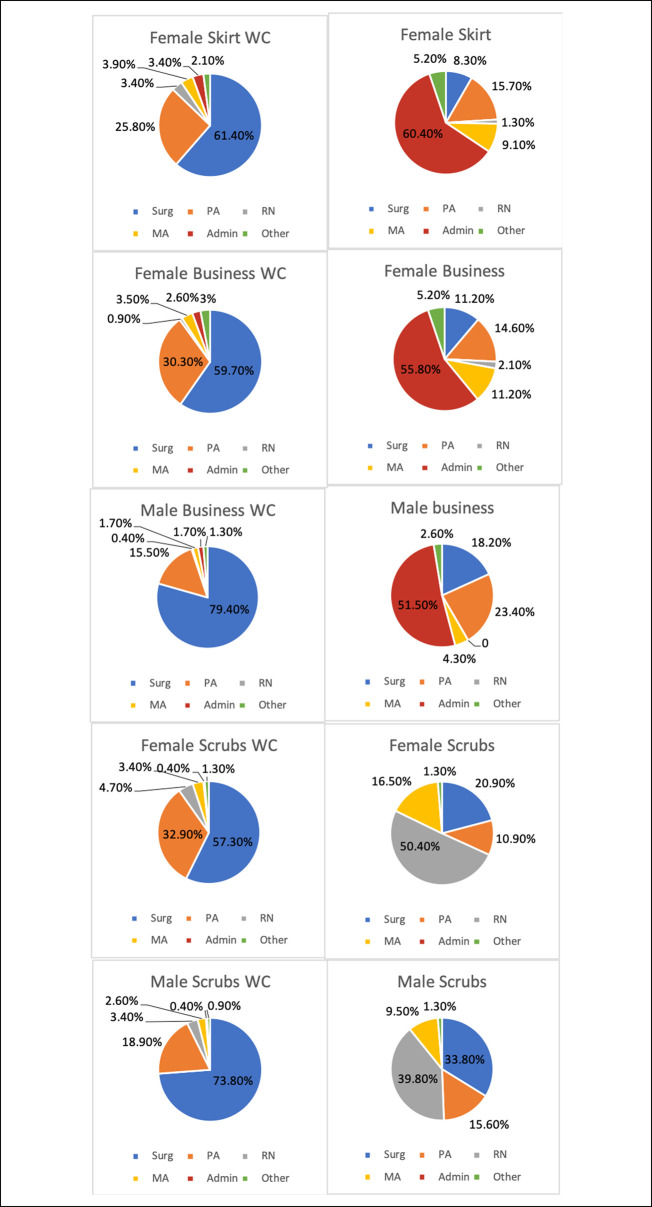
Pie charts depicting percentage rate of identification of role on healthcare team for each tested type of attire. Admin = hospital administrator; MA = medical assistant; PA = Physician Assistant; RN = Nurse; Surg = surgeon; WC = white coat.

Summative findings for the survey's identification portion are in Figure 3.

### Does Feminine Business Attire Affect Patient Perceptions of Four Realms of Surgical and Clinical Skills or Identification as a Surgeon?

Aggregated responses for the comparison questions can be found in Table 2. Patients found women in feminine attire with WCs significantly less likely to excel in performing the physical parts of surgery than the women in scrubs with WCs (8.4% to 24.3%, *P* = 0.001). Otherwise, women in feminine attire were rated comparably with women in a business attire and scrubs, as well as to men in a business attire for their competence, surgical outcomes, physical ability and trustworthiness.

**Table 2:** Summary of Paired Comparisons of Surgeon Traits Based on Attire

Comparison	Competence	Surgical Outcomes	Physical Components	Trustworthiness
Male scrubs	38 (17.2%)	25 (12.3%)	41 (20.2%)	28 (14.2%)
Male scrubs with WC	75 (33.9%)	59 (28.9%)	41 (20.2%)	40 (20.3%)
Same	108 (48.9%)	120 (58.8%)	121 (59.6%)	129 (65.5%)
Adjusted *P* value	**0.004**	**0.002**	1	0.55
Male business	21 (9.5%)	17 (8.1%)	14 (6.9%)	18 (9.4%)
Male business with WC	92 (41.8%)	82 (39.2%)	74 (36.6%)	50 (26.0%)
Same	107 (48.6%)	110 (52.6%)	114 (56.4%)	124 (64.6%)
Adjusted *P* value	**<0.001**	**<0.001**	**<0.001**	**0.001**
Female scrubs	24 (11.2%)	20 (9.8%)	33 (17.1%)	22 (11.1%)
Female scrubs with WC	97 (45.3%)	68 (33.2%)	51 (26.4%)	45 (22.6%)
Same	93 (43.5%)	117 (57.1%)	109 (56.5%)	132 (66.3%)
Adjusted *P* value	**<0.001**	**<0.001**	0.318	**0.043**
Female business	10 (4.8%)	7 (3.4%)	7 (3.4%)	10 (5.1%)
Female business with WC	108 (52.2%)	97 (46.9%)	89 (43.8%)	60 (30.6%)
Same	89 (43.0%)	103 (49.8%)	107 (52.7%)	126 (64.3%)
Adjusted *P* value	**<0.001**	**<0.001**	**<0.001**	**<0.001**
Female skirt	9 (4.3%)	5 (2.4%)	8 (4.1%)	6 (3.0%)
Female skirt with WC	110 (53.1%)	86 (41.1%)	85 (43.4%)	64 (32.0%)
Same	88 (42.5%)	118 (56.5%)	103 (52.6%)	130 (65.0%)
Adjusted *P* value	**<0.001**	**<0.001**	**<0.001**	**<0.001**
Male scrubs with WC	17 (8.2%)	8 (3.9%)	11 (5.6%)	6 (3.1%)
Female scrubs with WC	34 (16.3%)	26 (12.8%)	20 (10.1%)	28 (14.4%)
Same	157 (75.5%)	169 (83.3%)	167 (84.3%)	160 (82.5%)
Adjusted *P* value	0.12	**0.018**	0.452	**0.002**
Male business with WC	22 (10.6%)	19 (9.5%)	23 (11.6%)	10 (5.1%)
Female skirt with WC	26 (12.6%)	17 (8.5%)	16 (8.0%)	19 (9.6%)
Same	159 (76.8%)	163 (81.9%)	160 (80.4%)	168 (85.3%)
Adjusted *P* value	0.906	1	0.673	0.55
Male business with WC	19 (9.0%)	19 (9.3%)	24 (12.2%)	9 (4.6%)
Female business with WC	27 (12.7%)	14 (6.9%)	9 (4.6%)	22 (11.3%)
Same	166 (78.3%)	171 (83.8%)	163 (83.2%)	163 (84.0%)
Adjusted *P* value	0.906	1	0.089	0.156
Female business with WC	22 (10.9%)	21 (10.3%)	25 (12.4%)	19 (9.6%)
Female skirt with WC	29 (14.4%)	19 (9.3%)	14 (7.0%)	13 (6.6%)
Same	151 (74.8%)	164 (80.4%)	162 (80.6%)	165 (83.8%)
Adjusted *P* value	0.906	1	0.437	0.754
Female scrubs with WC	36 (16.7%)	43 (21.3%)	49 (24.3%)	23 (11.6%)
Female skirt with WC	59 (27.3%)	34 (16.8%)	17 (8.4%)	20 (10.1%)
Same	121 (56.0%)	125 (61.9%)	136 (67.3%)	156 (78.4%)
Adjusted *P* value	0.12	1	**<0.001**	0.76

WC = white coat

Significant *P* values (<0.05) are in bold type.

No difference was observed in how frequently women in feminine attire with a WC (61.4%) were identified as surgeons compared with women in other attire with WCs (scrubs: 57.3% and business attire: 59.7%; *P* > 0.999). For an attire without WCs, women in scrubs (20.9%) were identified as surgeons more frequently than women in both business (11.2%; *P* = 0.046) and feminine attire (8.3%; *P* = 0.003).

### Does Wearing a White Coat Affect Patient Perceptions of Four Realms of Surgical and Clinical Skills or Identification as a Surgeon?

Respondents uniformly ranked male and female orthopaedic surgeons wearing WCs on top of any attire as more competent and more likely to give a good surgical outcome than those without a WC (*P* < 0.005), although a large proportion of participants indicated that the presented models “seem the same” for each question (Table 2). Adding a WC over a business attire for both sexes increased perceived superiority in physical ability (male: 6.9% to 36.6%, *P* < 0.001; female business: 3.4% to 43.8%, *P* < 0.001; female skirt: 4.1% to 43%, *P* < 0.001), whereas adding a WC over scrubs did not change the perception of physical ability for either sex (male: 20.2% to 20.2%, *P* = 1; female: 17.1% to 26.4%, *P* = 0.318). Surgeons were perceived as more trustworthy when wearing a WC with male business attire (9.4% to 26%, *P* = 0.001), female scrubs (11.1% to 22.6%, *P* = 0.043), female business attire (5.1% to 30.6%, *P* < 0.001), and female skirt attire (3% to 32%, *P* < 0.001). Male orthopaedic surgeons in scrubs with and without WCs had similar trustworthiness ratings (14.2% to 20.3%, *P* = 0.55).

In the identification section, male and female models wearing a WC were unanimously identified as a surgeon more often than those in the same attire without a WC (all *P* < 0.001) (Table 3).

**Table 3:** Role Identification as a Surgeon for Matched Attire With and Without White Coats

Attire	White Coat	No White Coat	Adjusted *P* value
Female skirt	143 (61.4%)	19 (8.3%)	**<0.001**
Female business	138 (59.7%)	26 (11.2%)	**<0.001**
Female scrubs	134 (57.3%)	48 (20.9%)	**<0.001**
Male business	185 (79.4%)	42 (18.2%)	**<0.001**
Male scrubs	172 (73.8%)	78 (33.8%)	**<0.001**

Reported as N (%) who identified the picture as a surgeon. Significant *P* values (<0.05) are in bold type.

### How Readily Do Patients Identify Women as Surgeons as Compared to Men When an Identical Attire is Worn?

Significant differences were found in how respondents identified male and female surgeons wearing a comparable attire (Table 4). A man in any attire was identified as a surgeon more frequently than a woman comparably dressed. Notably, a man wearing a business attire with a WC was identified as a surgeon 79.4% of the time, whereas a woman wearing a WC with business pants or skirt was identified as a surgeon 59.7% or 61.4% of the time, respectively (both *P* < 0.001). A woman in scrubs without a WC was identified as a nurse by 50.4% of respondents and as a surgeon by only 20.9%. In comparison, a man in scrubs without a WC identified as a nurse 39.8% and surgeon 33.8% of the time (*P* = 0.031). Wearing a WC with scrubs increased the rate of identification as a surgeon for women to 57.3% and for men to 73.8%. Both men and women in a business attire (including skirts) without WCs were most frequently identified as hospital administrators.

**Table 4:** Role Identification as a Surgeon for Matched Attire by Sex

Attire	Female	Male	Adjusted *P* value
Business with white coat	138 (59.7%)	185 (79.4%)	**<0.001**
Skirt with white coat	143 (61.4%)	—	**<0.001**^a^
Business, no white coat	26 (11.2%)	42 (18.2%)	0.252
Skirt, no white coat	19 (8.3%)	—	**0.025**^a^
Scrubs with white coat	134 (57.3%)	172 (73.8%)	**0.003**
Scrubs, no white coat	48 (20.9%)	78 (33.8%)	**0.025**

Reported as N (%) who identified the picture as a surgeon. Significant *P* values (<0.05) are in bold type.

a *P* values from female skirt compared with male business counterpart

### Are Female Surgeons in Any Attire Perceived Differently than Male Surgeons?

In the comparison section, respondents frequently indicated no preference between the presented pairs of photos. The option of “they seem the same” was chosen most frequently for all comparisons except for the three questions assessing competence for female attire with and without a WC. There, the image of the model wearing a WC was chosen most frequently. Women in scrubs with a WC were judged more likely to give a good surgical outcome and more trustworthy than men in the same attire (12.8% versus 3.9%, *P* = 0.018; 14.4% versus 3.1%, *P* = 0.002, respectively).

## Discussion

This study of 238 patients from sport medicine and foot and ankle orthopaedic subspecialty clinics demonstrated that surgeon attire affects patient perceptions of surgeon skill and that attire influences whether a provider is identified as a surgeon.

In this study, respondents uniformly ranked physicians wearing WCs over any attire as more competent and more likely to give a good surgical outcome than those without. Surgeons wearing a WC over any type of business attire, including skirts, were deemed more likely to excel in the physical parts of surgery; for surgeons in scrubs, no statistical difference was observed between those with or without a WC. In many recent studies, patients have indicated a general preference for doctors to wear WCs. In a multicenter study by Petrilli et al^1^ of nonsurgical patients, over half of the patients agreed or strongly agreed that doctors should wear a WC when seeing patients in the hospital or in their office. Lands found that patients in an orthopaedic hand clinic consistently gave the highest ranking to male and female surgeons wearing a WC in seven patient-perceived qualities.^5^ In our study, no comparisons existed where a greater number of respondents preferred the model without a WC to one with a WC. Therefore, for most patients who indicated a preference, a WC seems to increase association with positive traits.

Although the WC may be viewed favorably by patients, there has been concern about WCs as potential fomites that contribute to the spread of bacteria among hospitalized patients. In 2007, hospitals in the United Kingdom began a “bare below the elbows” policy to address the spread of healthcare-associated infections, barring long sleeves, traditional WCs, and wristwatches, among other attire.^11^ However, WCs may not be the culprit because a 2011 study reported that after 8 hours of wear, no differences were observed in bacterial colony counts or methicillin resistant Staphylococcus aureus (MRSA) growth from newly laundered short-sleeve scrubs versus infrequently laundered WCs.^12^ Furthermore, some British authors have raised concern that the hospital-mandated loss of the WC has lowered doctors' recognizability among other healthcare workers.^13^ In our study, men and women wearing a business attire without a WC were most frequently identified as hospital administrators, whereas those in the same attire paired with a WC were identified as administrators less than 3% of the time. Particularly in the inpatient setting, where representatives from administrative departments such as billing, registration, or patient relations are more likely to interact with patients, the WC seems to be a key factor in recognizability as a physician.

In our investigation, the rate of identification as a surgeon was markedly higher for all tested types of attire when a WC was worn versus not worn. However, gender-based differences were found. Although women could boost their recognizability as a surgeon for any given attire by wearing a WC, they were still more frequently identified as nonphysician healthcare workers than men in comparable attire. Misidentification as a nurse or PA is a commonplace occurrence for many female surgeons, particularly in orthopaedics, where only 4% of the AAOS members are women.^2^ This can cause strain and confusion in the physician-patient relationship because patients may anchor to their initial judgments and have difficulty treating the female surgeon as the leader of their surgical team. Frequent instances of misidentification and patient-expressed doubt about their role as the surgeon are not only frustrating for the surgeon but may dovetail with a phenomenon known as “imposter syndrome,” in which a person feels persistently inadequate and underperforming, despite external achievements and accomplishments to the contrary. An American College of Physicians position article from 2018 identified imposter syndrome as a notable challenge for women in medicine and a contributor to disproportionately low numbers of women physicians achieving academic advancement and serving in leadership positions.^14^ Additional works focusing on high-achieving careers such as medicine and STEM (science, technology, engineering, and math) fields have demonstrated that women in high-achieving fields are disproportionately affected by imposter syndrome.^15,16,17,18^ Our data provide credence to this often-experienced misidentification and may serve to support interventions for imposter syndrome targeted toward women in medicine.

Orthopaedics has long held a reputation as a surgical subspecialty reliant on, even dominated by, physical strength.^19^ The relative dearth of women in the field may be related to the perception that too much physical strength was required, as suggested by 74% of the RJOS members surveyed by Rohde et al.^2^ Carnes et al have described that male-gendered stereotypes of “agentic” traits (e.g., assertiveness and technical prowess) versus female-gendered stereotypes of “communal” traits (e.g., empathy and good listening skills) contribute to gaps in professional achievement between men and women in medicine, academia, and research.^10,20,21^ We hypothesized that feminine attire, such as skirts and dresses, would lower patient perceptions in skills more associated with men, such as physical strength, but increase rankings of more “communal” skills, such as trustworthiness. In our study, patients rated women in skirts markedly less likely to excel in the physical parts of surgery than women in scrubs, suggesting that dressing in unisex, nongendered attire gave female surgeons the outward appearance of more strength than if they wore a skirt. Feminine attire did not seem to give an advantage in appearing more trustworthy in this study. Other recent orthopaedic-focused attire surveys have included women in skirts, but as their only “business attire” model, precluding comparison with other less overtly feminine attire. Furthermore, although comparisons within each sex allowed a preferred attire to be identified, previous studies have not published direct comparisons between men and women orthopaedic surgeons.^3-5^ One previous study of the importance of the orthopaedic doctors' appearance from Scotland did not include any female models.^6^

Another important finding we noted was that in the “comparison” portion of our survey, the option of “they seem the same” was chosen most frequently for all comparisons except for the three questions assessing competence for female attire with and without a WC. There, the option with the model wearing a WC was chosen most frequently. Although subtle, this suggests that the presence of a WC is more important for conferring competence for women than for men. It also implies that the WC is a stronger marker for competence than the other three tested surgeon traits of likelihood to give a good surgical outcome, physical strength in the operating room, or trustworthiness.

Our study has several strengths. To our knowledge, it includes the largest number of respondents for any orthopaedic-specific attire study conducted in the United States. We recruited patients from both sport medicine and foot and ankle, neither subspecialty having previous published attire studies. We recruited patients from two subspecialties (Sports Medicine and Foot and Ankle) for which there are not previously published attire studies, with a male and female surgeon from each subspecialty.

Our study has several limitations. One of the most critical is that our models were young, Caucasian, and nonobese. We matched the age and racial demographics and body habitus of our two models to allow the attire to be the primary variable under investigation. The authors do acknowledge that the influence of age, race, and body habitus are likely among the most important but least-investigated factors that may alter patient perceptions of their surgeons. Fox et al^22^ published a study of dermatology patients in Miami that included both Caucasian and black physician models in their survey and noted respondents were more likely to prefer professional attire for black physicians than white physicians and for women more than men. Even so, a reply to the editor lamented that the authors did not discuss the sex and racial bias in more detail.^23^ The total number of non-Caucasian orthopaedic surgeons has been increasing but is still just over 15% of practicing orthopaedic surgeons based on the 2018 AAOS Census Survey.^24^ Obese job candidates may be less likely to be hired and assessed as having less leadership skill than their normal weight peers.^25^ The age of included models has varied in recent studies, but it is reasonable to think that patients may judge older physicians to be more competent or be more likely to identify them as a senior member of the team. Future studies that examine these factors will help to further delineate bias in orthopaedics and identify means by which to combat these biases.

Ours is one of the first orthopaedics-oriented attire studies to span multiple subspecialties and to include patients from both male and female orthopaedic surgeons. However, we omitted other orthopaedic subspecialty patients who may have unique underpinnings to their perceptions of surgeons. For example, many orthopaedic trauma patients do not first meet their orthopaedic surgeons in the outpatient setting, but rather are cared for by whomever is on call that day. Alternatively, orthopaedic oncology visits often involve discussion of sensitive topics that patients may prefer to discuss with a surgeon dressed more formally. Future studies spanning the breadth of subspecialties in orthopaedics would be valuable.

There are also likely regional differences in attire standards that were not fully captured in this single center study, which reflects the environment at a Midwestern large academic institution. Unlike most other published attire studies, we did not include any models in casual attire. It has not been our experience that surgeons attempt to wear casual attire such as t-shirts and jeans to conduct regular clinical duties. Furthermore, all previously cited studies that tested casual attire found it to be consistently and overwhelmingly disliked by patients, a finding we deemed unnecessary to investigate again.

In conclusion, attire influences patient perceptions of their orthopaedic surgeons. Female surgeons who wear feminine business attire instead of scrubs may be rated less able to perform the physical work of operating but are otherwise rated comparably with their male peers. By magnitude of response, the WC was more important for women than men in conferring competence. Surgeons wearing WCs over any attire were deemed more competent and more likely to give a good surgical outcome than those without. Few other gender-based differences in surgeon skill were identified. Men are more readily identified as surgeons than women when wearing a similar attire. On the basis of this study, men may choose to don a WC to improve patient perceptions of competence and ability to provide a good surgical outcome. Women orthopaedic surgeons may experience a higher likelihood of being identified and judged as a competent surgeon if they wear a WC and viewed as more likely to excel in physical tasks in the operating room if they don scrubs instead of business attire.

## Supplementary Material

SUPPLEMENTARY MATERIAL
