# Supplementary material for: The Effect of an Orthopaedic Surgeon's Attire on Patient Perceptions of Surgeon Traits and Identity: A Cross-Sectional Survey
Source: J Am Acad Orthop Surg Glob Res Rev. 2020 Aug 3;4(8):e20.00097. doi: 10.5435/JAAOSGlobal-D-20-00097 (PMC7418901; doi:10.5435/JAAOSGlobal-D-20-00097)
Supplement: SUPPLEMENTARY MATERIAL [file jagrr-4-e20.00097-s001.pdf]

Demographics

Thank you for participating in our survey. Your decision to participate and your responses will in no way affect the care you receive. Responses are recorded anonymously.

Please select your age:

- Under 18
- 18-25
- 26-34
- 35-49
- 50-65
- 66-80
- 81+

I am:

- Male
- Female
- Other
- Prefer not to say

Please select the highest level of education you achieved:

- Before high school
- High school graduate
- Some college
- Bachelor's degree
- Master's degree
- Doctorate degree
- Prefer not to say

Please select your race/ethnicity (select all that apply)

- White
- Black or African American
- American Indian or Alaska Native
- Asian
- Native Hawaiian or Pacific Islander
- Hispanic
- Other
- Prefer not to say

Please select which best describes the area where you live:

- Urban
- Suburban
- Rural

Please select which best describes your appointment today:

- New patient
- Post-operative visit
- Returning patient
- Emergency room follow-up
- Other

Intro Pictures

Next, you are going to see pictures of someone you may encounter in the hospital or clinic. For each picture presented, you will be asked to identify their most likely role in the hospital or clinic. Select the answer that is closest to your best guess. You may use the same answer multiple times.

Pictures

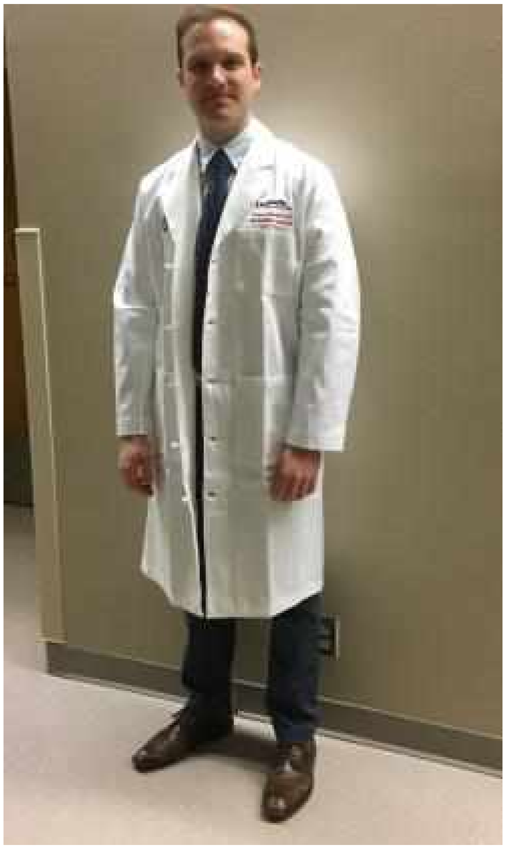

What do you think is this person's role on the healthcare team? Please select your best guess from the options below.

- Surgeon (doctor)
- Physician Assistant/PA (non-MD who sees patients under the supervision of a doctor)
- Nurse
- Medical Assistant/MA (helps clinic run smoothly by rooming patients, taking vitals, etc.)
- Hospital Administrator
- Other

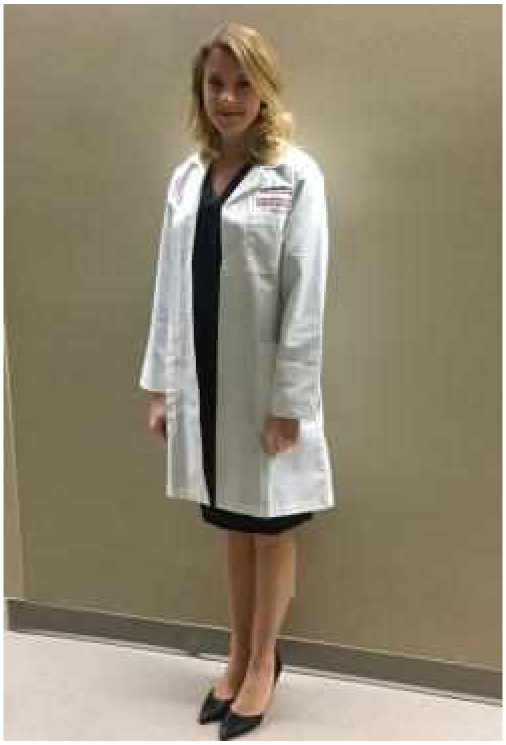

What do you think is this person's role on the healthcare team? Please select your best guess from the options below.

- Surgeon (doctor)
- Physician Assistant/PA (non-MD who sees patients under the supervision of a doctor)
- Nurse
- Medical Assistant/MA (helps clinic run smoothly by rooming patients, taking vitals, etc.)
- Hospital Administrator
- Other

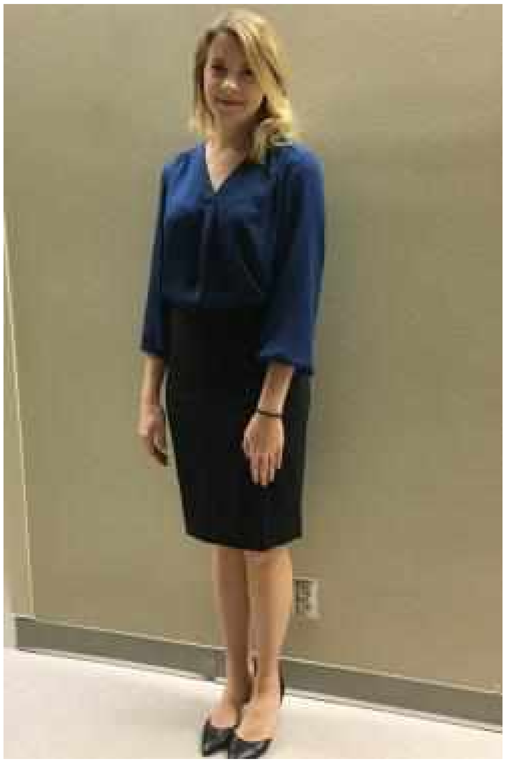

What do you think is this person's role on the healthcare team? Please select your best guess from the options below.

- Surgeon (doctor)
- Physician Assistant/PA (non-MD who sees patients under the supervision of a doctor)
- Nurse
- Medical Assistant/MA (helps clinic run smoothly by rooming patients, taking vitals, etc.)
- Hospital Administrator
- Other

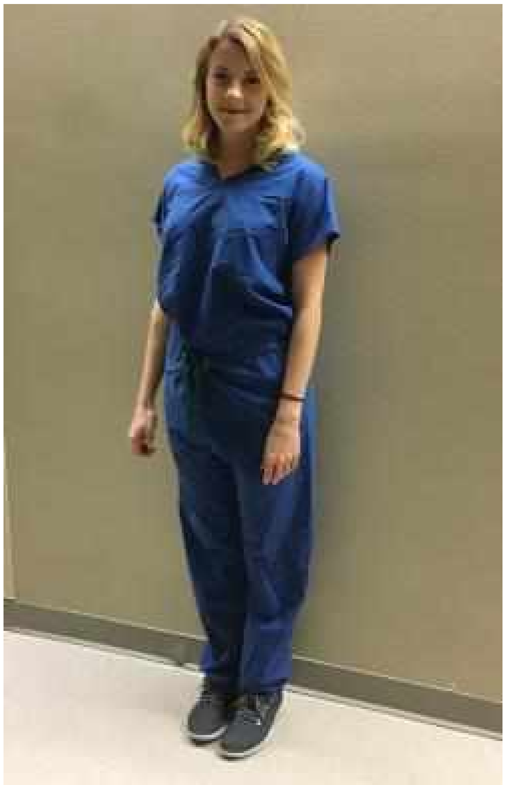

What do you think is this person's role on the healthcare team? Please select your best guess from the options below.

- Surgeon (doctor)
- Physician Assistant/PA (non-MD who sees patients under the supervision of a doctor)
- Nurse
- Medical Assistant/MA (helps clinic run smoothly by rooming patients, taking vitals, etc.)
- Hospital Administrator
- Other

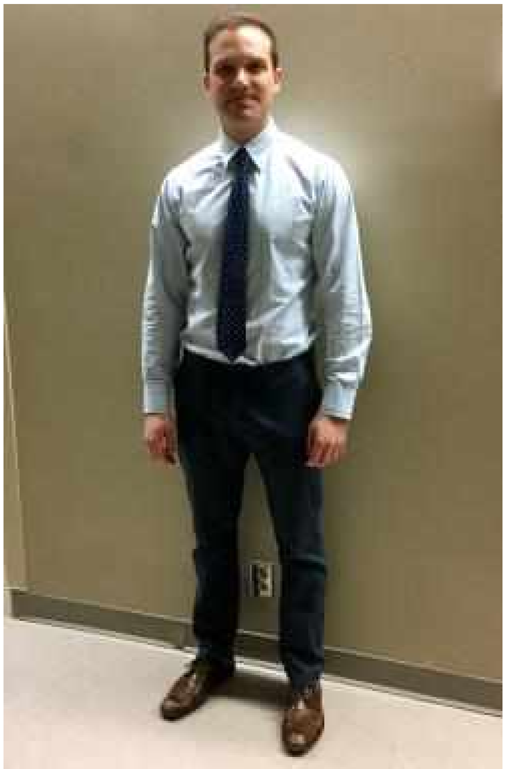

What do you think is this person's role on the healthcare team? Please select your best guess from the options below.

- Surgeon (doctor)
- Physician Assistant/PA (non-MD who sees patients under the supervision of a doctor)
- Nurse
- Medical Assistant/MA (helps clinic run smoothly by rooming patients, taking vitals, etc.)
- Hospital Administrator
- Other

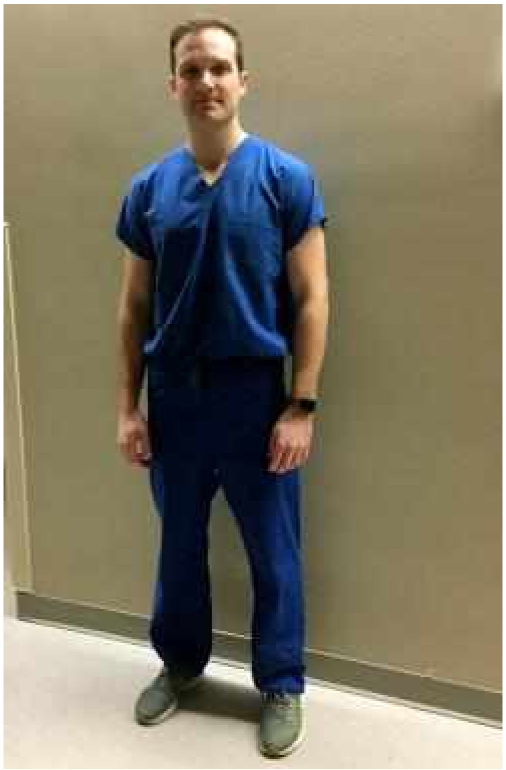

What do you think is this person's role on the healthcare team? Please select your best guess from the options below.

- Surgeon (doctor)
- Physician Assistant/PA (non-MD who sees patients under the supervision of a doctor)
- Nurse
- Medical Assistant/MA (helps clinic run smoothly by rooming patients, taking vitals, etc.)
- Hospital Adminstrator
- Other

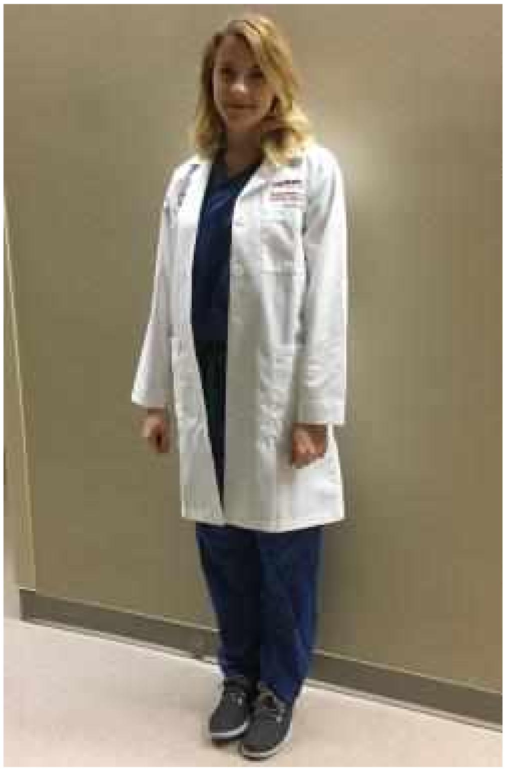

What do you think is this person's role on the healthcare team? Please select your best guess from the options below.

- Surgeon (doctor)
- Physician Assistant/PA (non-MD who sees patients under the supervision of a doctor)
- Nurse
- Medical Assistant/MA (helps clinic run smoothly by rooming patients, taking vitals, etc.)
- Hospital Administrator
- Other

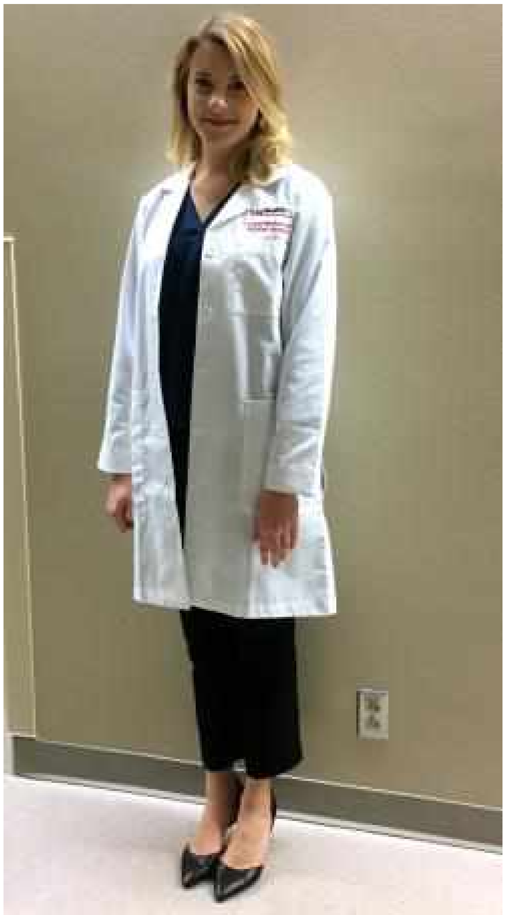

What do you think is this person's role on the healthcare team? Please select your best guess from the options below.

- Surgeon (doctor)
- Physician Assistant/PA (non-MD who sees patients under the supervision of a doctor)
- Nurse
- Medical Assistant/MA (helps clinic run smoothly by rooming patients, taking vitals, etc.)
- Hospital Administrator
- Other

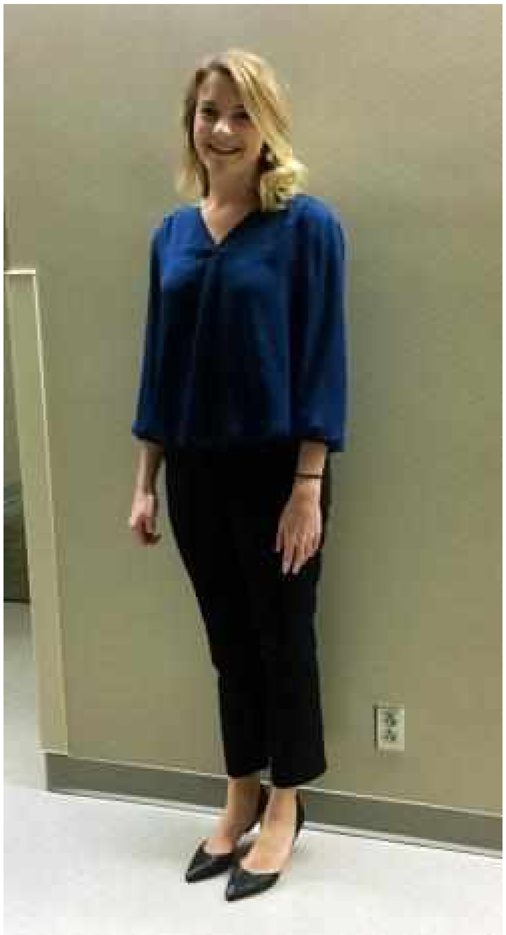

What do you think is this person's role on the healthcare team? Please select your best guess from the options below.

- Surgeon (doctor)
- Physician Assistant/PA (non-MD who sees patients under the supervision of a doctor)
- Nurse
- Medical Assistant/MA (helps clinic run smoothly by rooming patients, taking vitals, etc.)
- Hospital Administrator
- Other

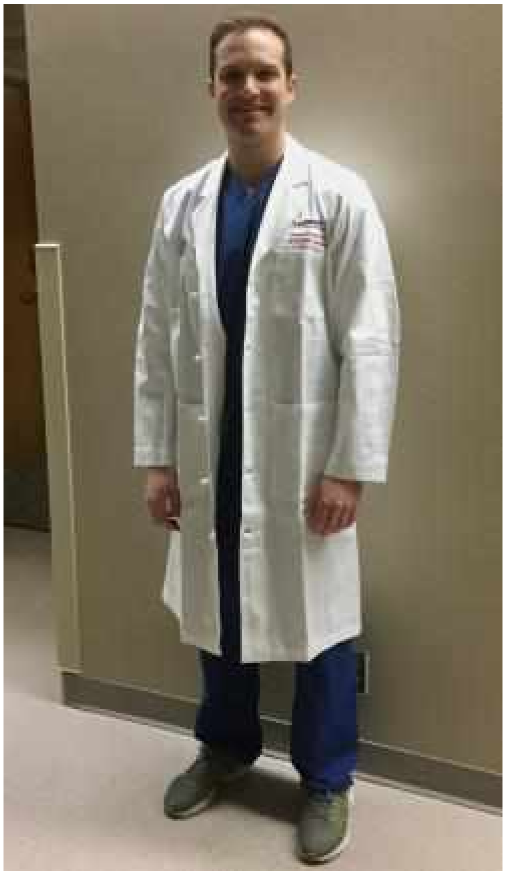

What do you think is this person's role on the healthcare team? Please select your best guess from the options below.

- Surgeon (doctor)
- Physician Assistant/PA (non-MD who sees patients under the supervision of a doctor)
- Nurse
- Medical Assistant/MA (helps clinic run smoothly by rooming patients, taking vitals, etc.)
- Hospital Administrator
- Other

Intro Competence

In the third part of this survey, imagine that you are having an appointment in the orthopedic surgery clinic with the surgeon pictured. You will be asked to pick which surgeon fits with certain characteristics as listed on the slide.

This first section will ask you to pick a surgeon based on who you think is more **competent**.

Competence

Imagine that you are having a clinic visit with the orthopedic surgeon shown below. Which provider seems more **competent**?

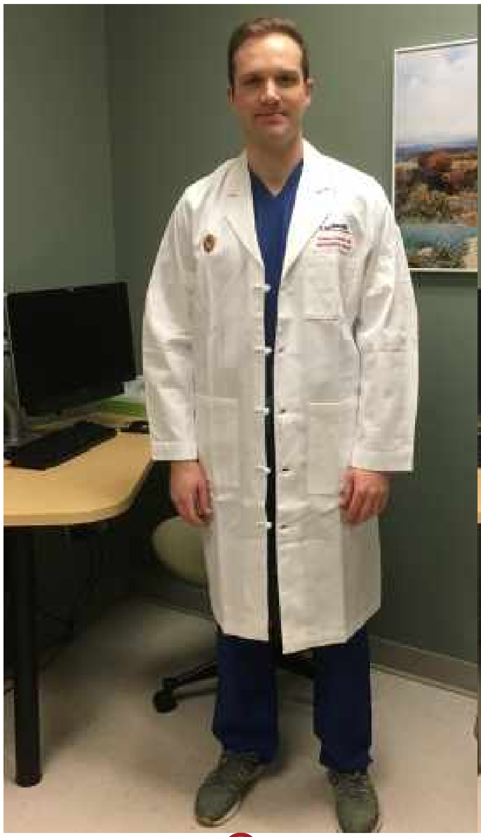

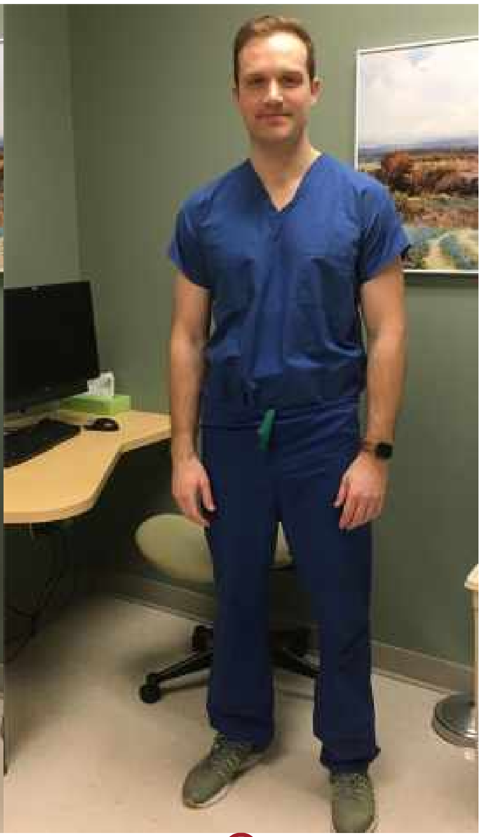

They seem the same

Imagine that you are having a clinic visit with the orthopedic surgeon shown below. Which provider seems more **competent**?

They seem the same

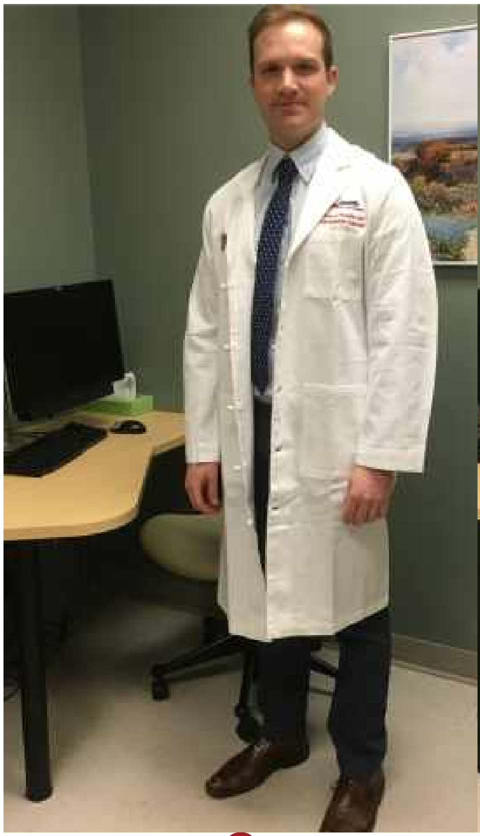 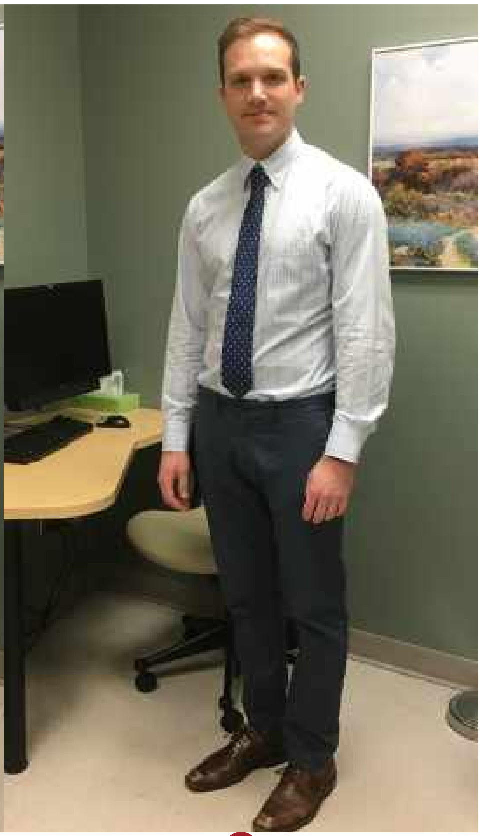

Imagine that you are having a clinic visit with the orthopedic surgeon shown below. Which provider seems more **competent**?

They seem the same

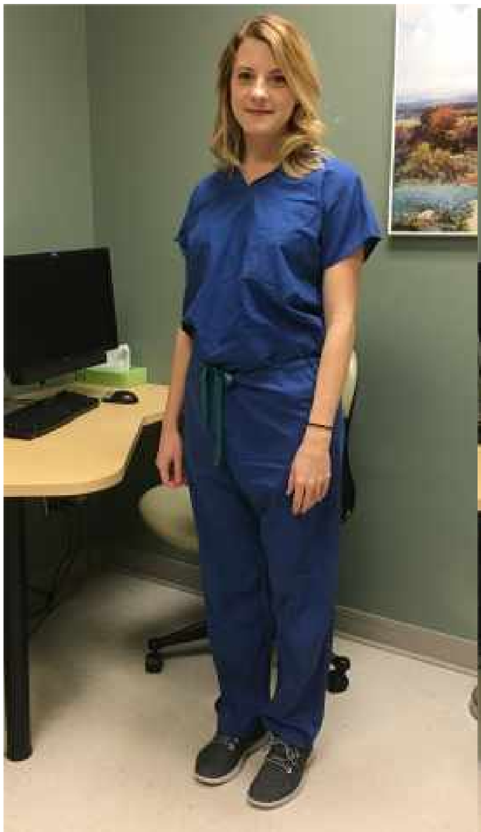 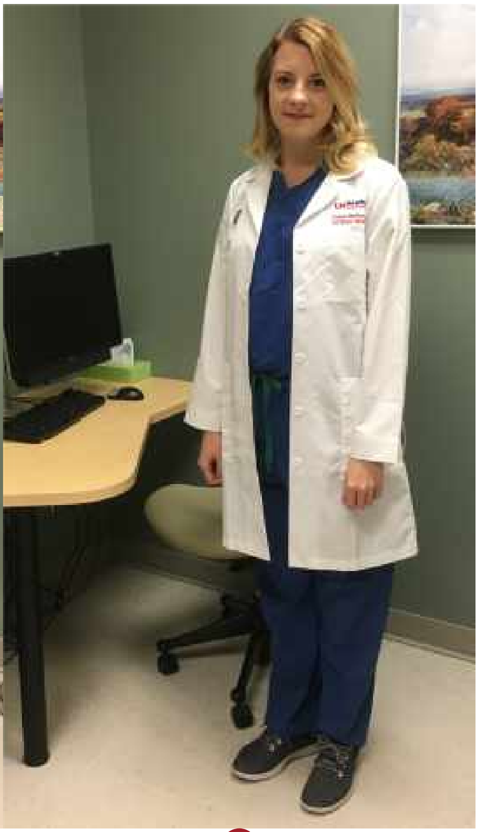

Imagine that you are having a clinic visit with the orthopedic surgeon shown below. Which provider seems more **competent**?

They seem the same

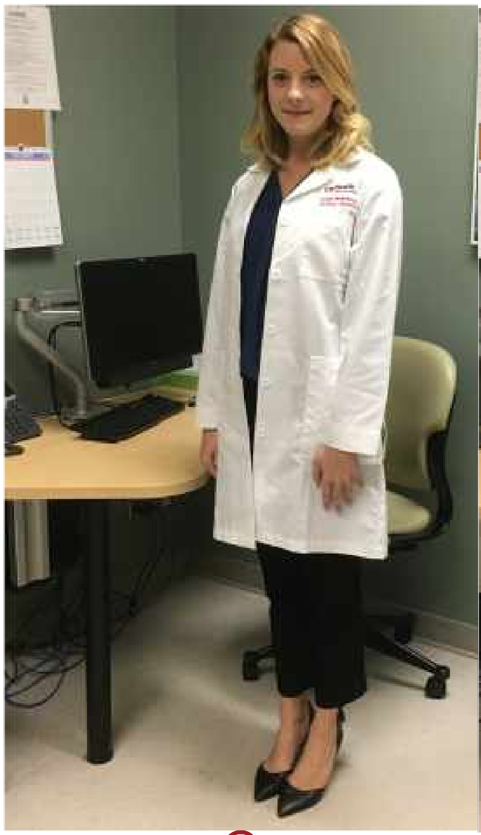

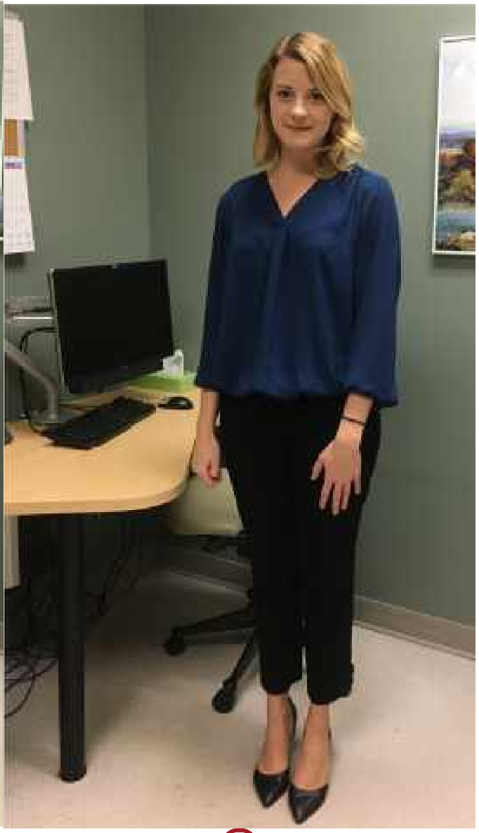

Imagine that you are having a clinic visit with the orthopedic surgeon shown below. Which provider seems more **competent**?

They seem the same

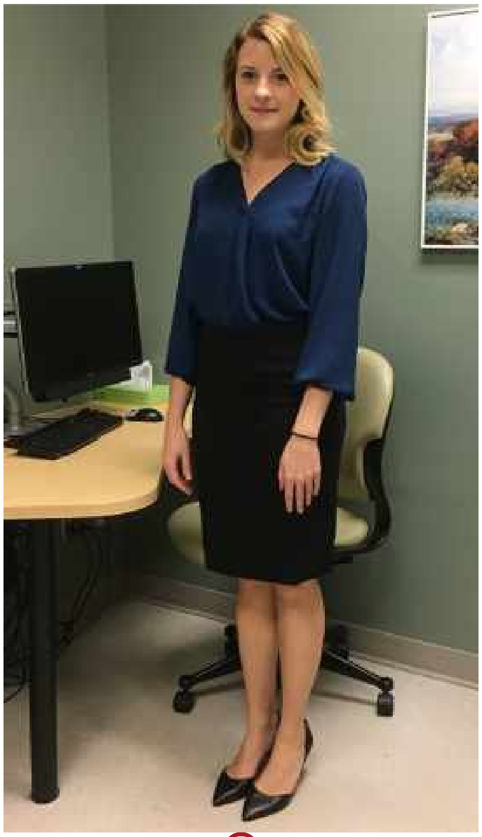

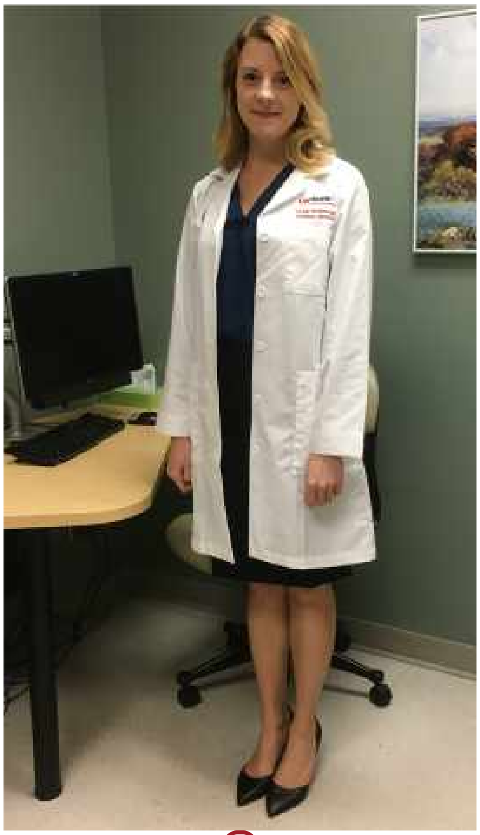

Imagine that you are having a clinic visit with the orthopedic surgeon shown below. Which provider seems more **competent**?

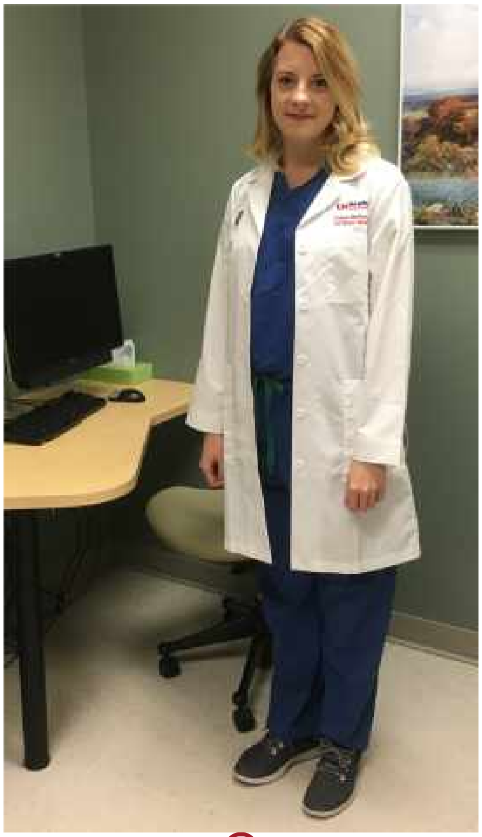

They seem the same

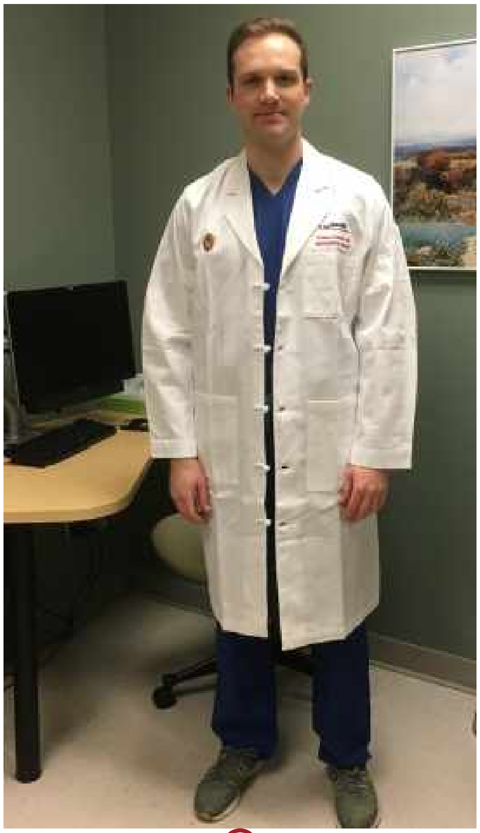

Imagine that you are having a clinic visit with the orthopedic surgeon shown below. Which provider seems more **competent**?

They seem the same

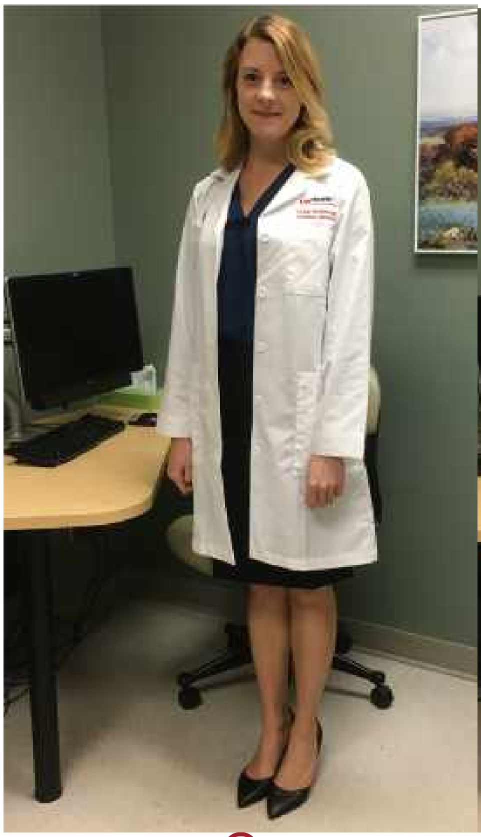

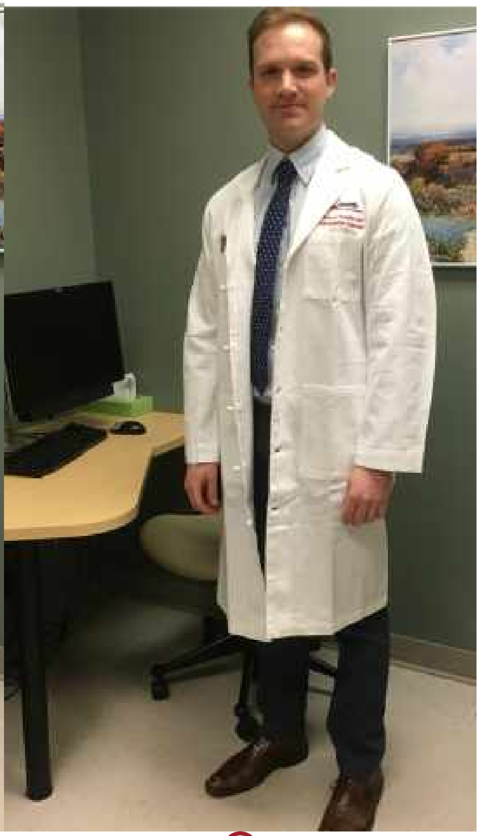

Imagine that you are having a clinic visit with the orthopedic surgeon shown below. Which provider seems more **competent**?

They seem the same

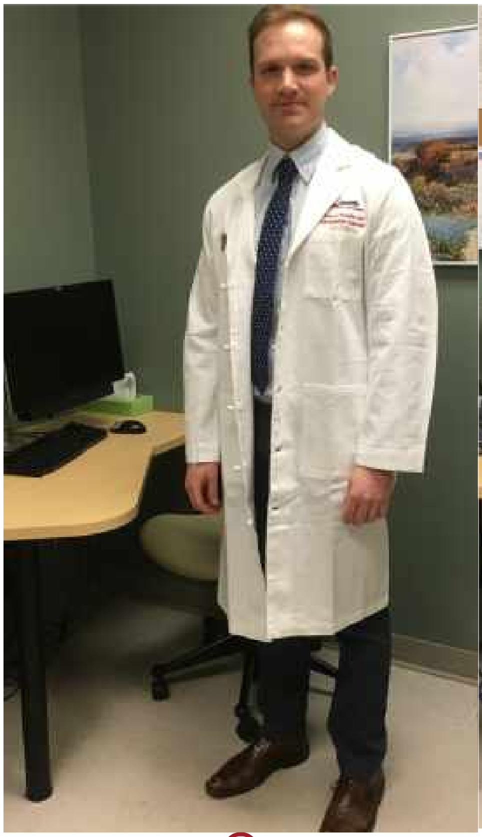

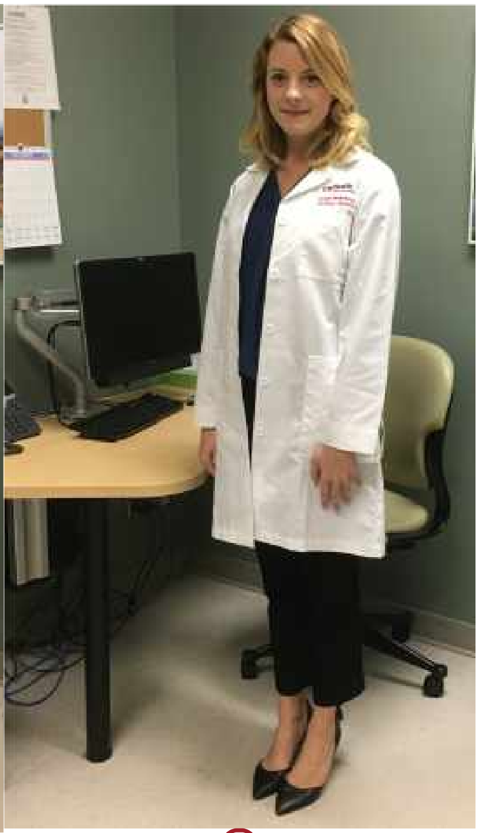

Imagine that you are having a clinic visit with the orthopedic surgeon shown below. Which provider seems more **competent**?

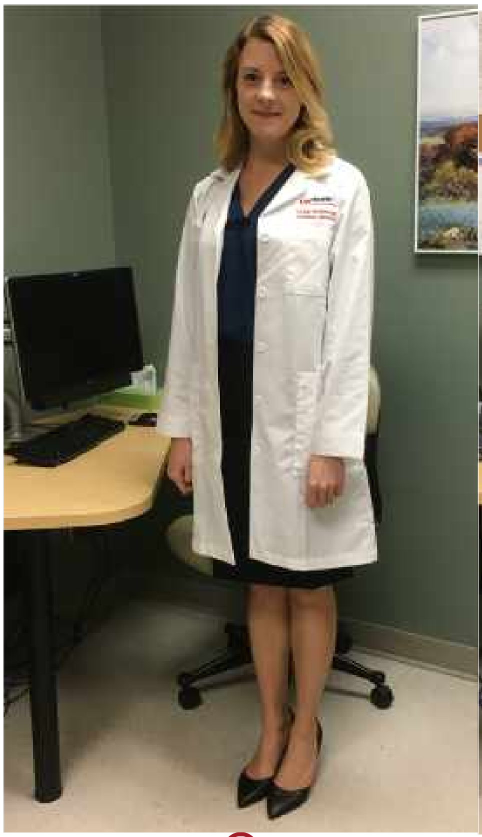

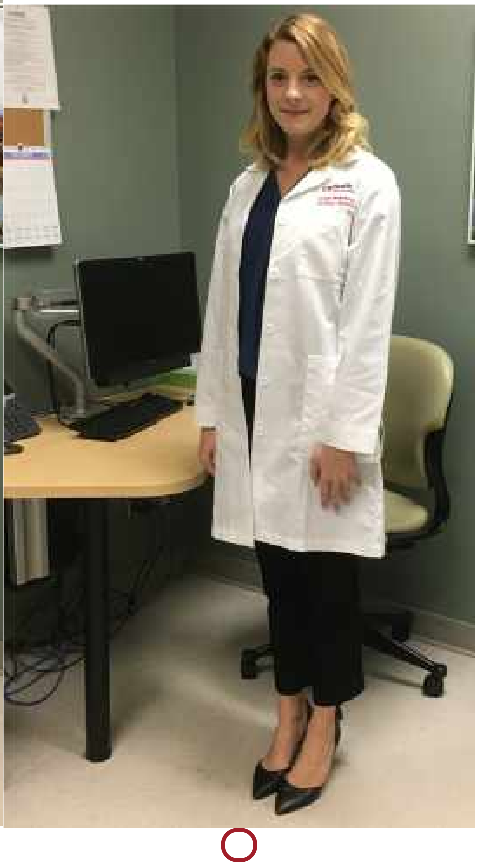

They seem the same

Imagine that you are having a clinic visit with the orthopedic surgeon shown below.
Which provider seems more **competent**?

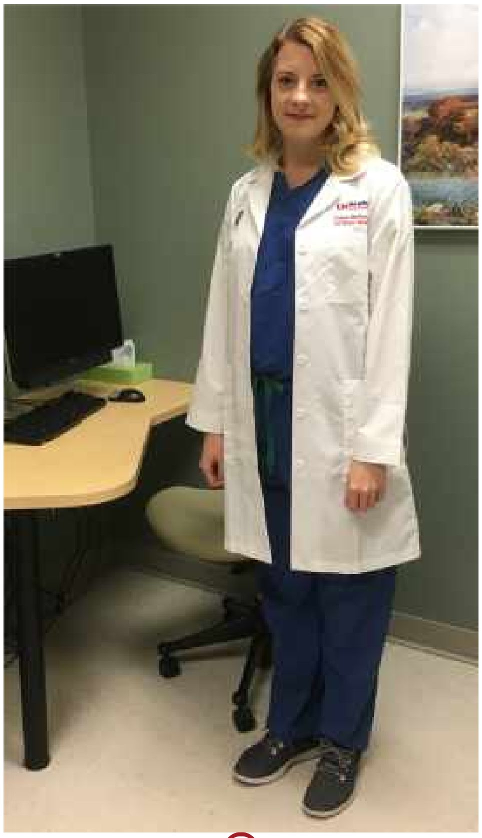

They seem the same

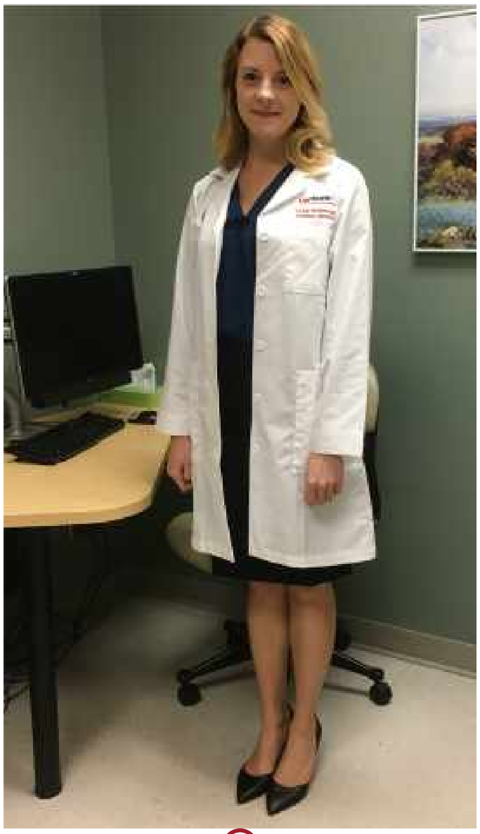

○ ○ ○

Imagine that you are having a clinic visit with the orthopedic surgeon shown below.
Which provider seems more **competent**?

They seem the same

○

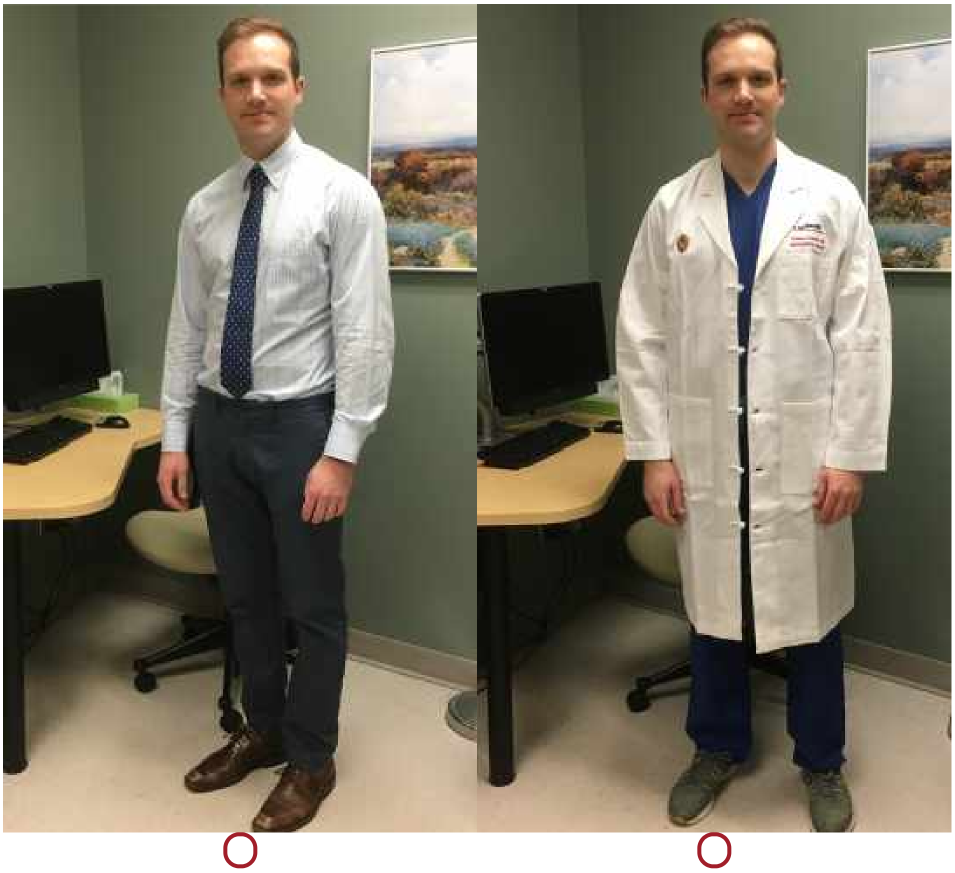

○ ○

Intro Surgical Outcome

This second section will ask you to pick a surgeon based on who you think will give you a **better surgical outcome**.

Surgical Outcomes 1

Imagine that you are having a clinic visit with the orthopedic surgeon shown below. Which surgeon seems more likely to give a **better surgical outcome**?

They seem the same

○

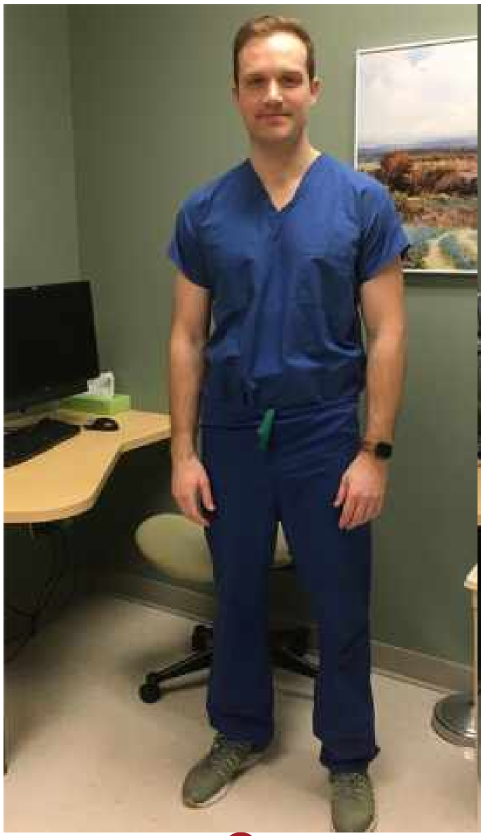

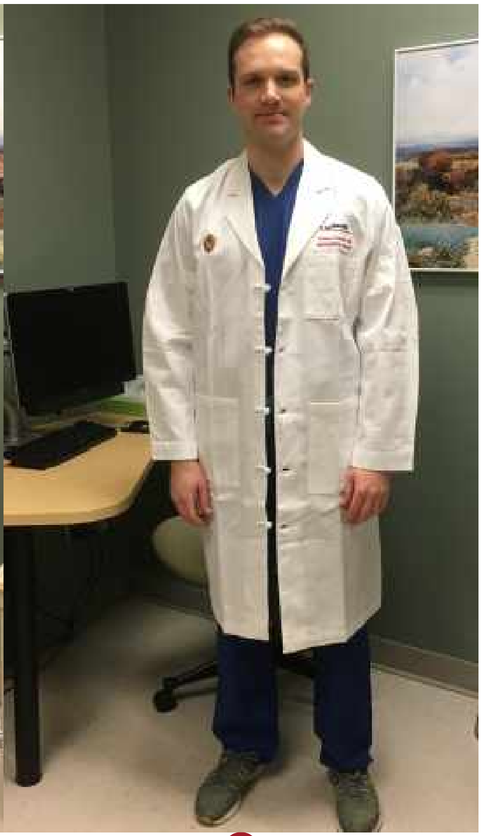

Imagine that you are having a clinic visit with the orthopedic surgeon shown below. Which surgeon seems more likely to give a **better surgical outcome**?

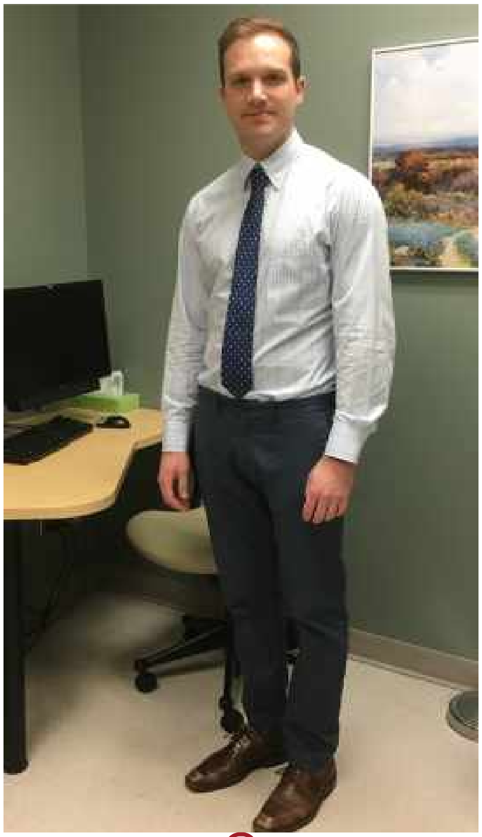

They seem the same

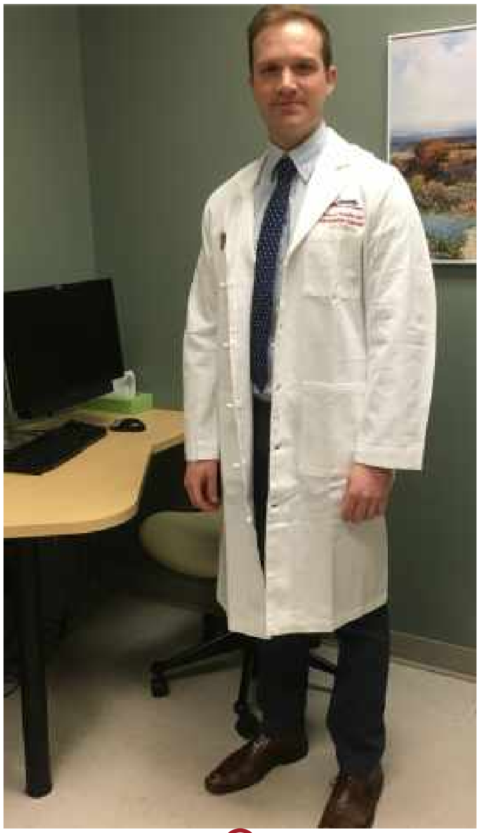

Imagine that you are having a clinic visit with the orthopedic surgeon shown below. Which surgeon seems more likely to give a **better surgical outcome**?

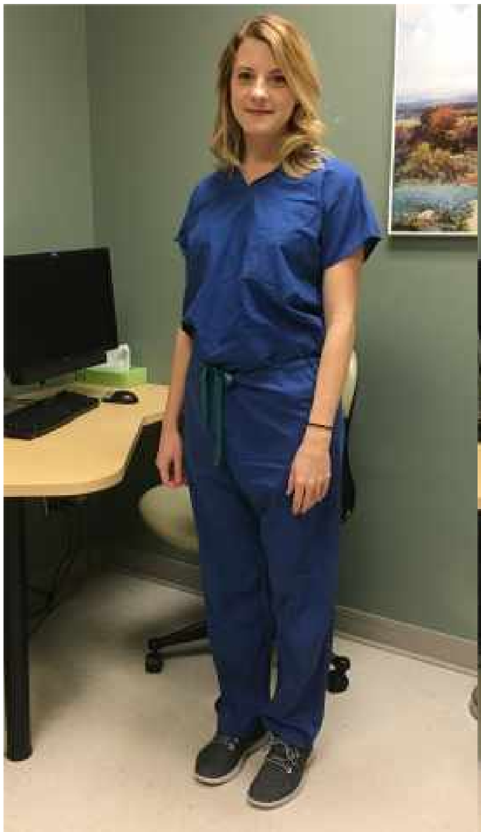

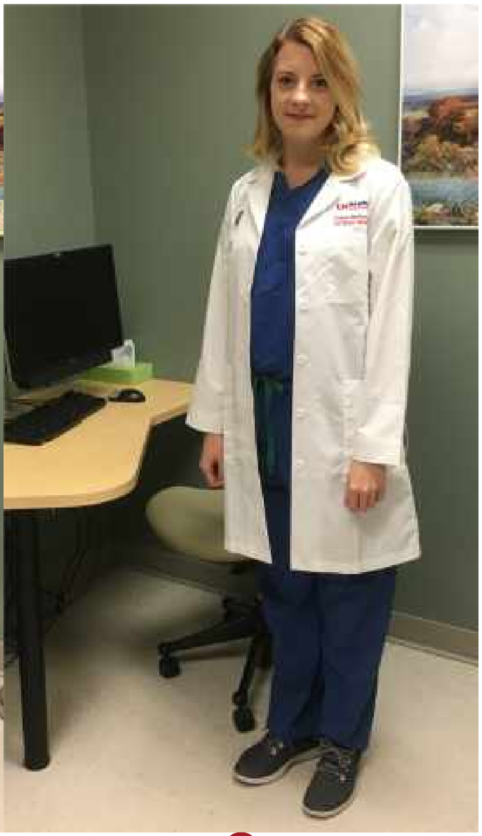

They seem the same

Imagine that you are having a clinic visit with the orthopedic surgeon shown below. Which surgeon seems more likely to give a **better surgical outcome**?

They seem the same

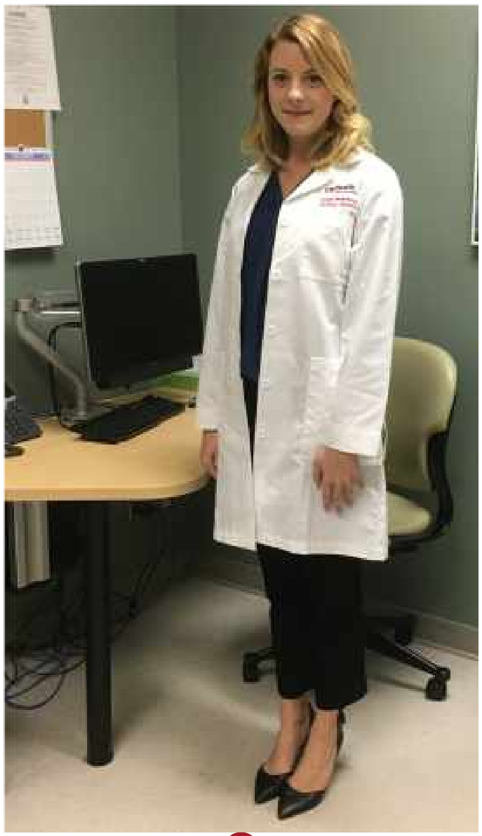

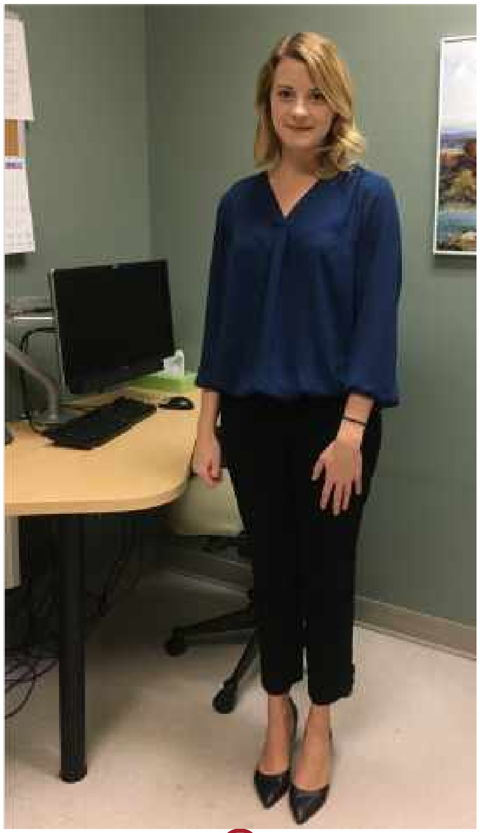

Imagine that you are having a clinic visit with the orthopedic surgeon shown below. Which surgeon seems more likely to give a **better surgical outcome**?

They seem the same

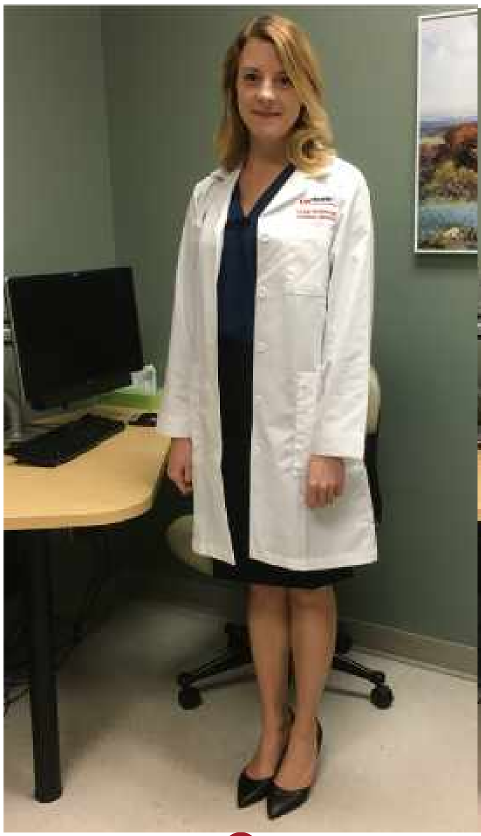

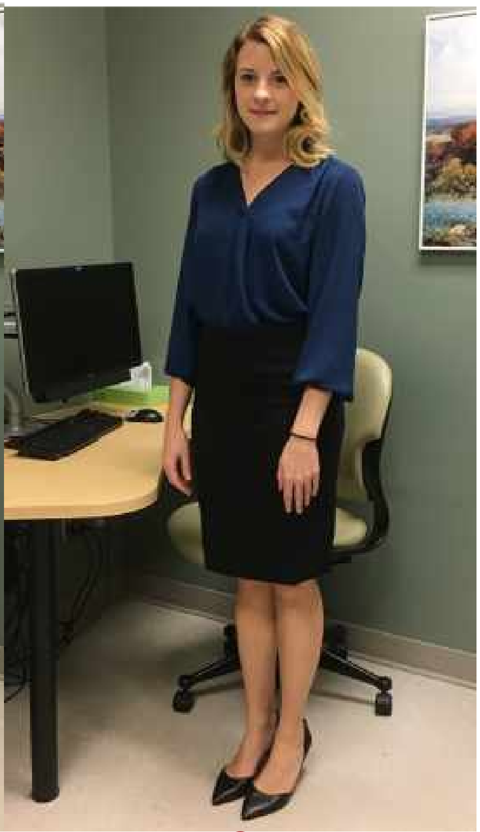

Imagine that you are having a clinic visit with the orthopedic surgeon shown below. Which surgeon seems more likely to give a **better surgical outcome**?

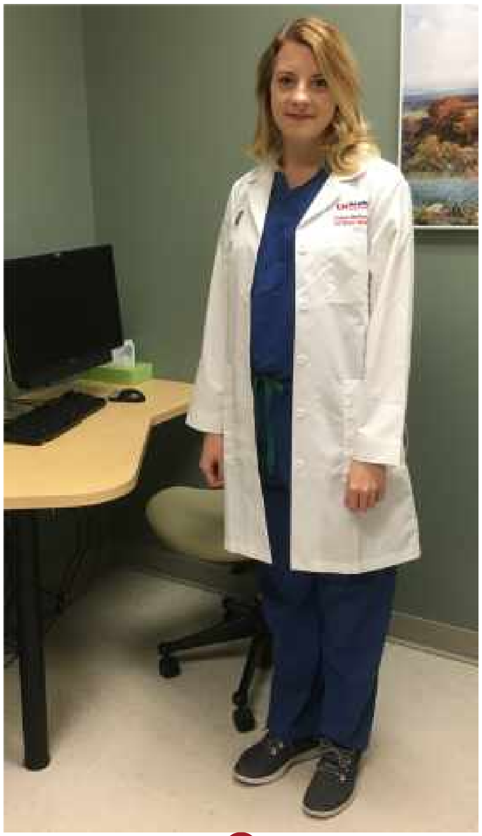

They seem the same

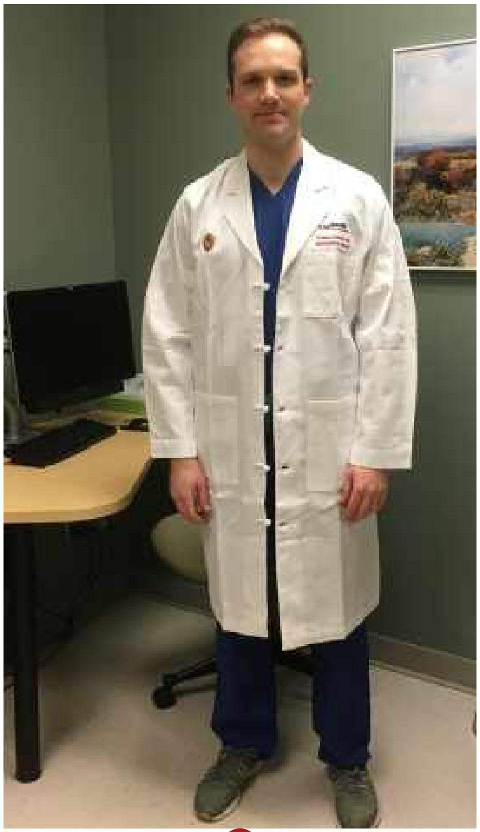

Imagine that you are having a clinic visit with the orthopedic surgeon shown below. Which surgeon seems more likely to give a **better surgical outcome**?

They seem the same

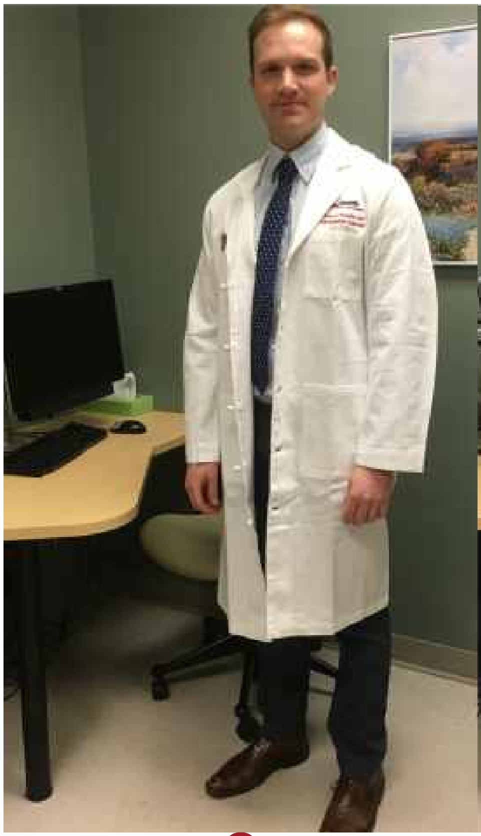
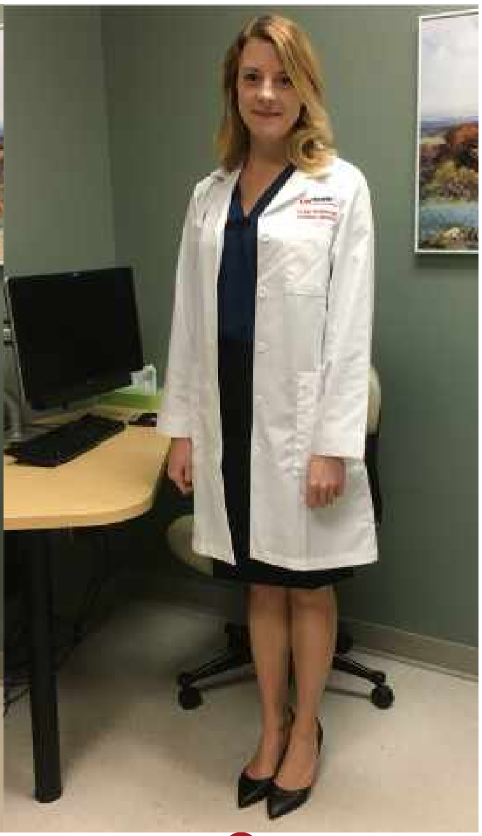

Imagine that you are having a clinic visit with the orthopedic surgeon shown below. Which surgeon seems more likely to give a **better surgical outcome**?

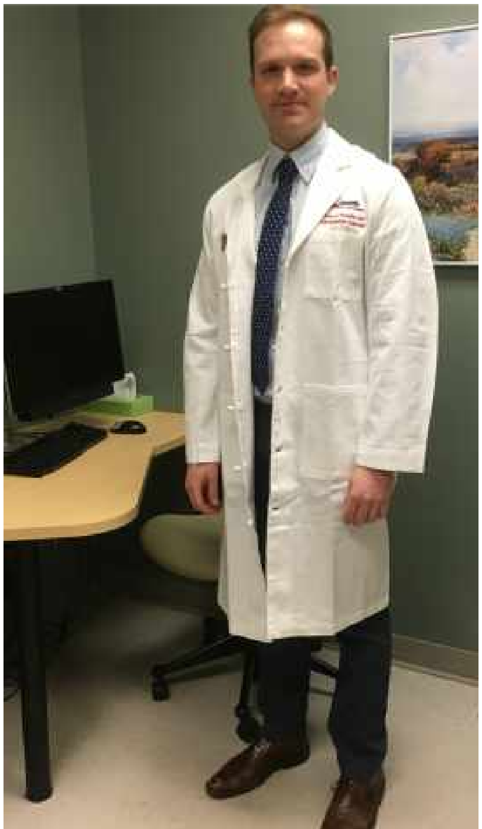

They seem the same

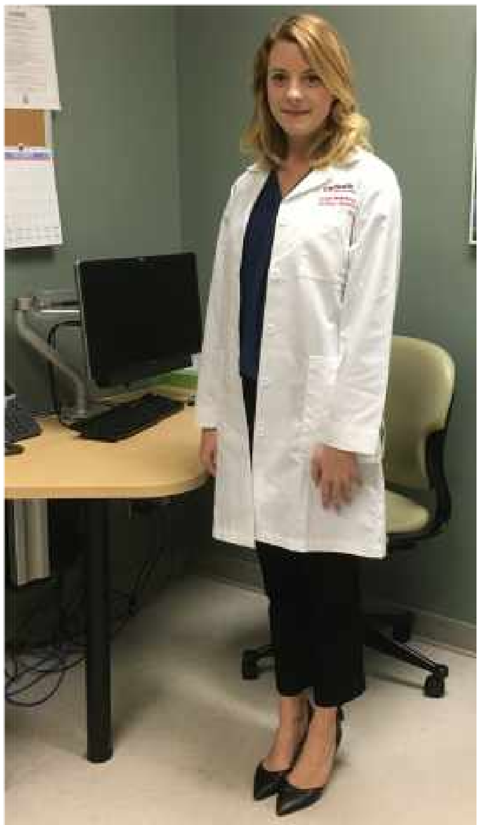

Imagine that you are having a clinic visit with the orthopedic surgeon shown below. Which surgeon seems more likely to give a **better surgical outcome**?

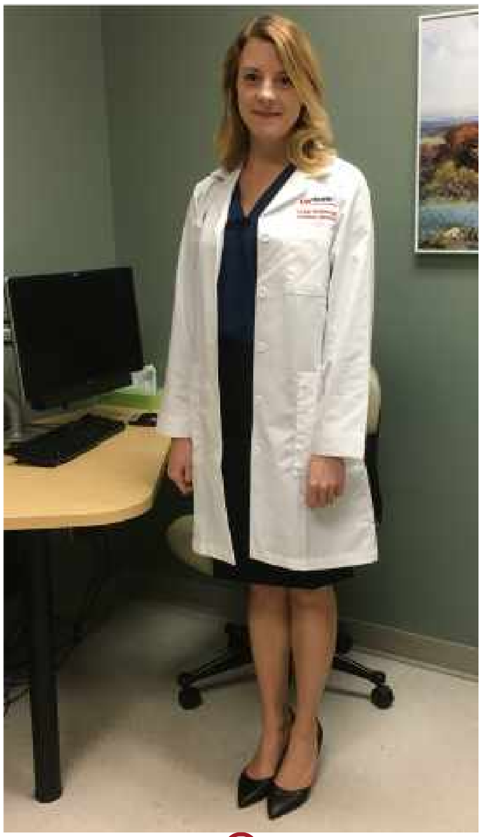

They seem the same

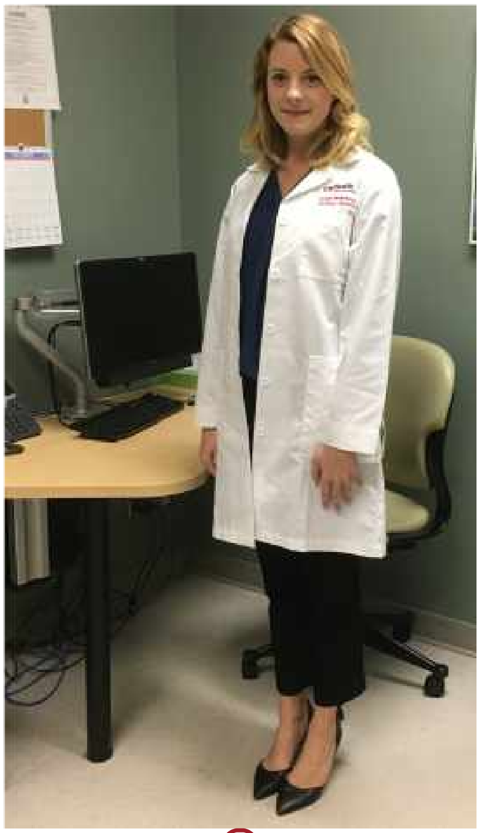

Imagine that you are having a clinic visit with the orthopedic surgeon shown below. Which surgeon seems more likely to give a **better surgical outcome**?

They seem the same

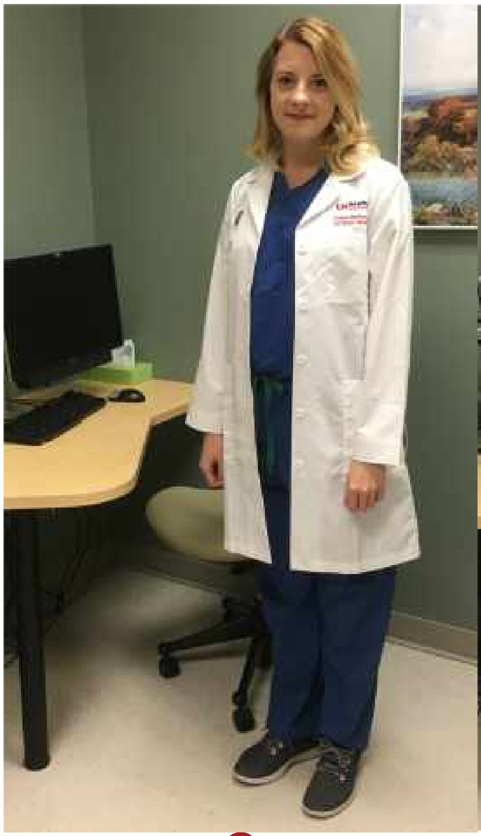

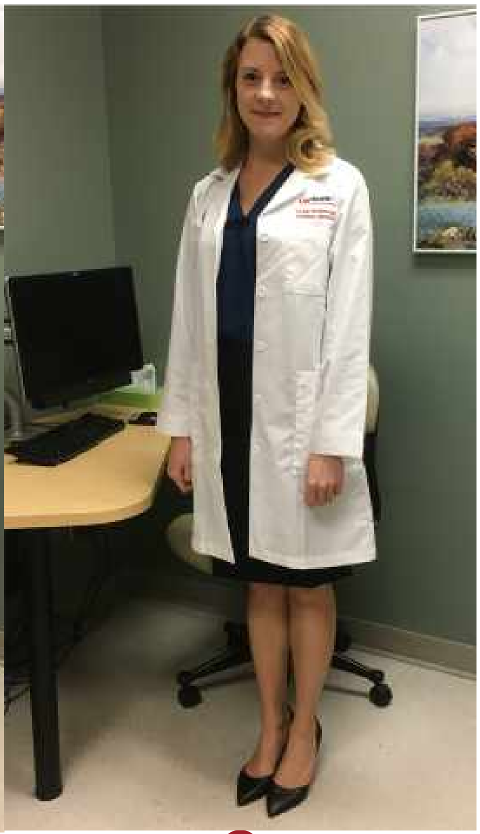

○ ○

Imagine that you are having a clinic visit with the orthopedic surgeon shown below. Which surgeon seems more likely to give a **better surgical outcome**?

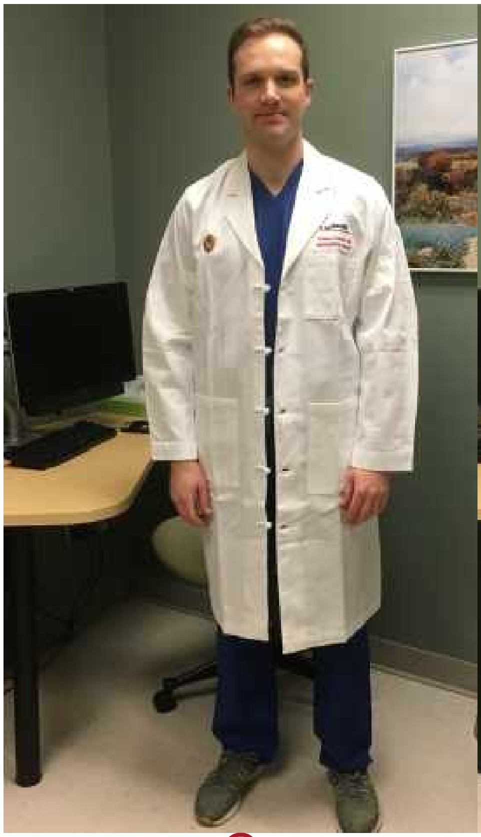

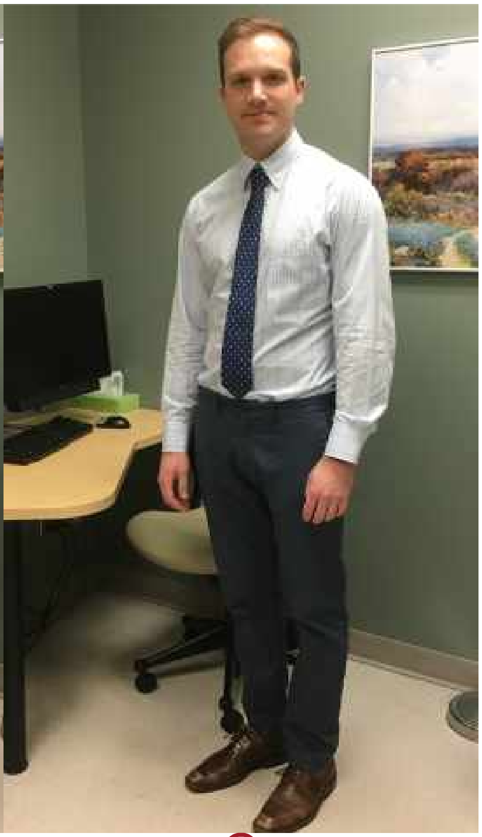

They seem the same

○ ○ ○

Intro Physical Aspects

This third section will ask you to pick a surgeon based on who you think is more likely to **excel in performing the physical parts of surgery**.

Physical Aspects 1

Imagine that you are having a clinic visit with the orthopedic surgeon shown below. Which provider seems more likely to **excel in performing the physical parts of surgery**?

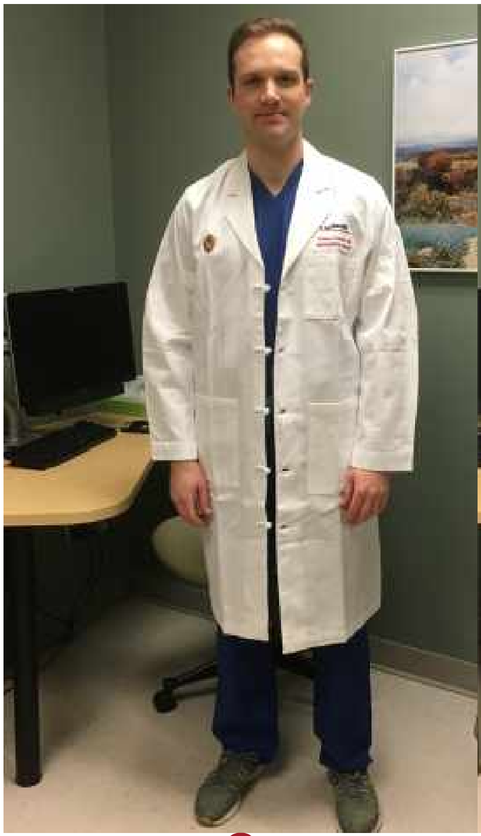

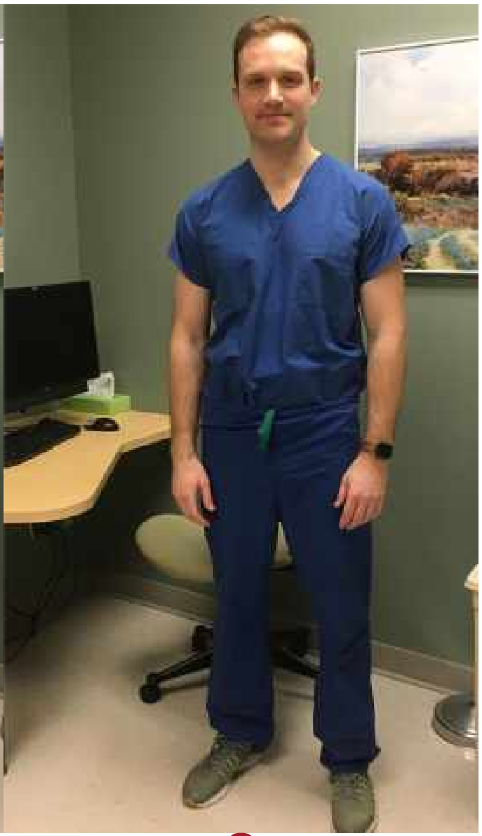

They seem the same

Imagine that you are having a clinic visit with the orthopedic surgeon shown below. Which provider seems more likely to **excel in performing the physical parts of surgery**?

They seem the same

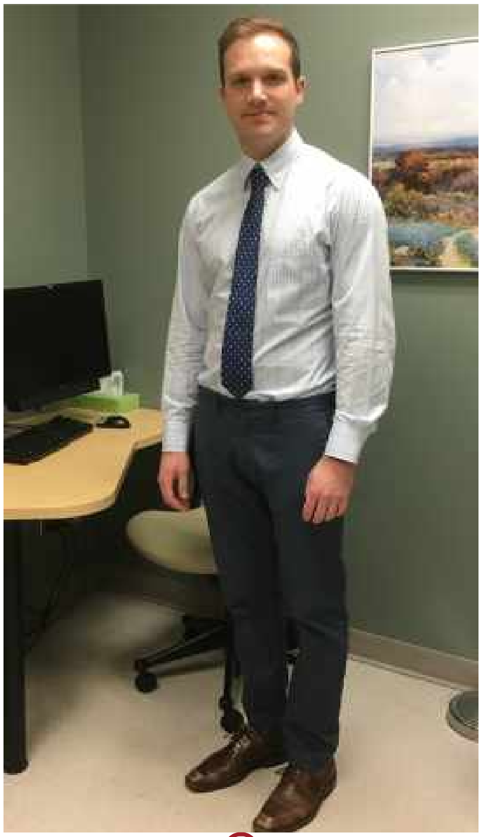

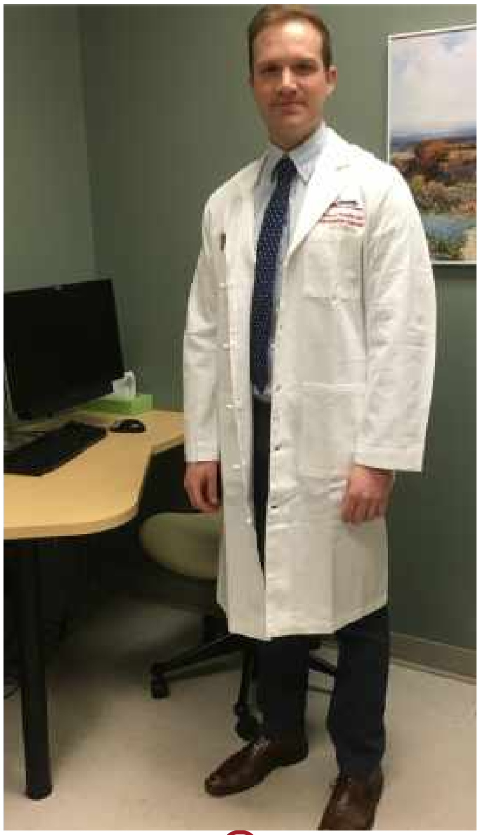

Imagine that you are having a clinic visit with the orthopedic surgeon shown below. Which provider seems more likely to **excel in performing the physical parts of surgery**?

They seem the same

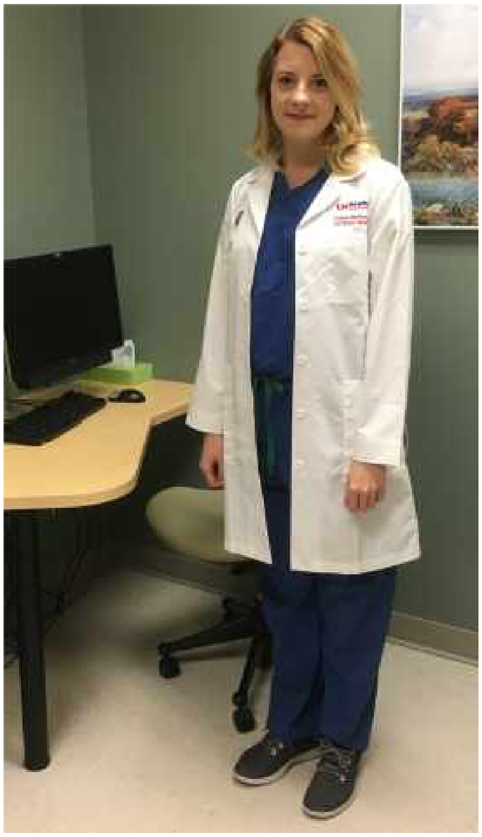

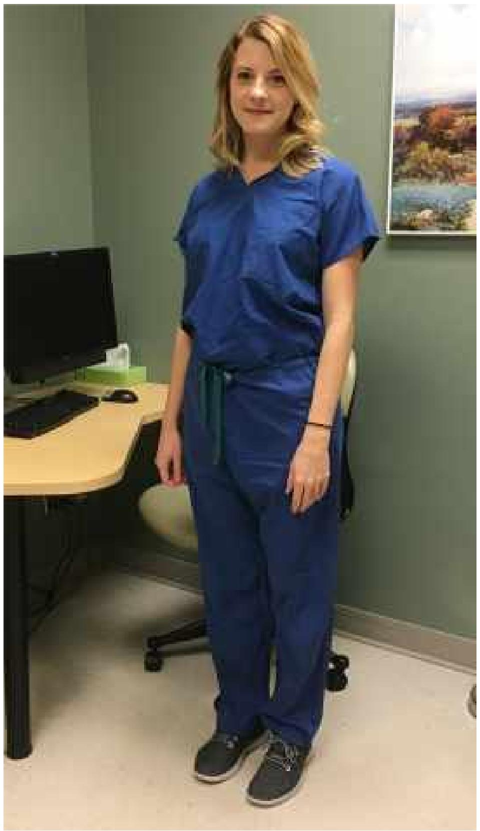

Imagine that you are having a clinic visit with the orthopedic surgeon shown below. Which provider seems more likely to **excel in performing the physical parts of surgery**?

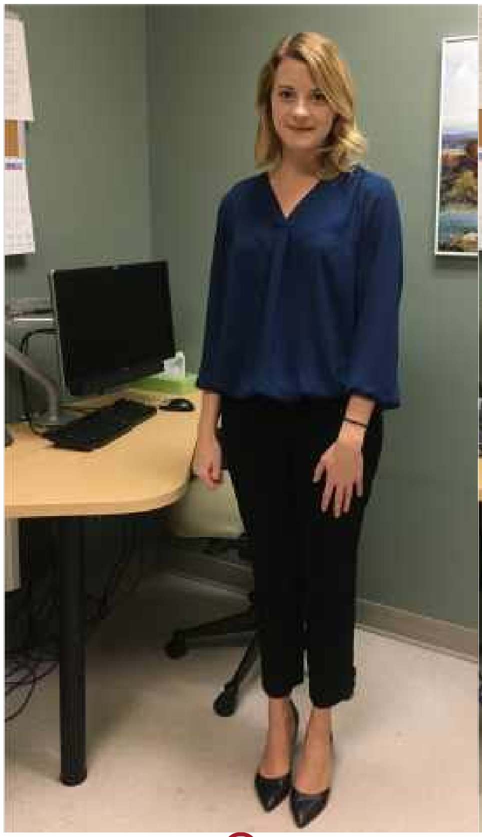

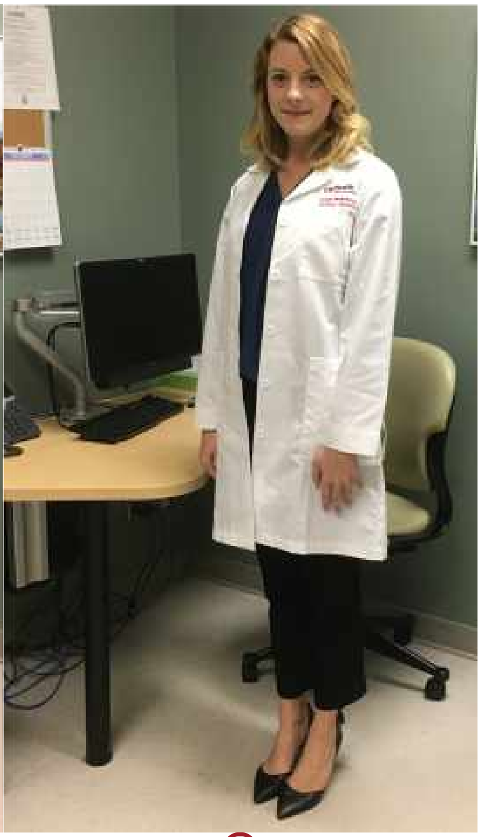

They seem the same

Imagine that you are having a clinic visit with the orthopedic surgeon shown below. Which provider seems more likely to **excel in performing the physical parts of surgery**?

They seem the same

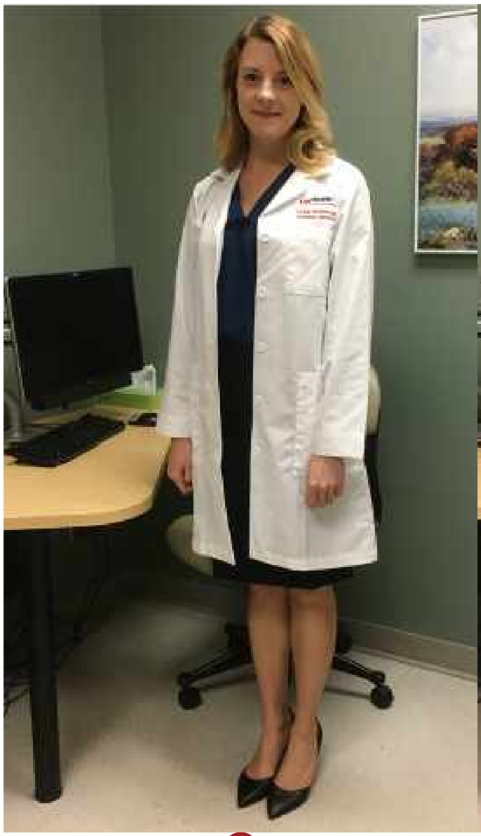

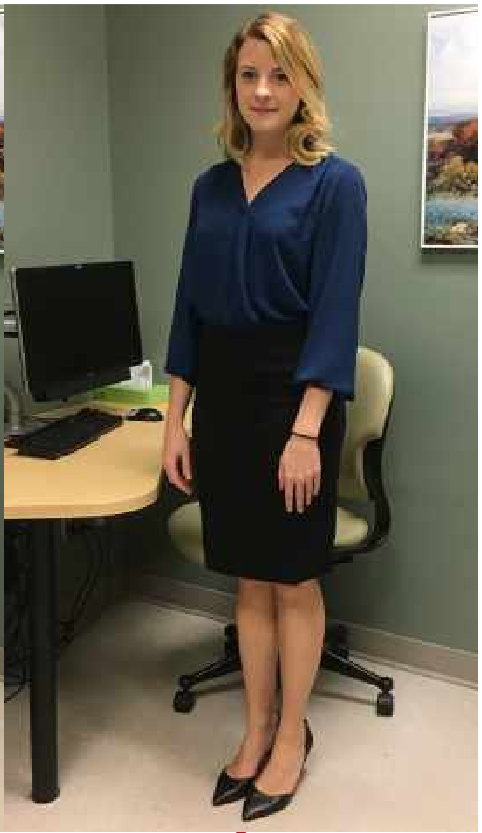

Imagine that you are having a clinic visit with the orthopedic surgeon shown below. Which provider seems more likely to **excel in performing the physical parts of surgery**?

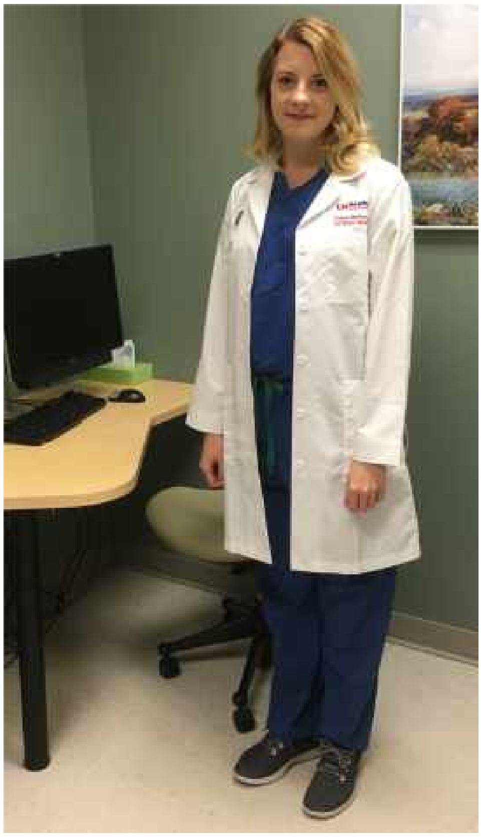

They seem the same

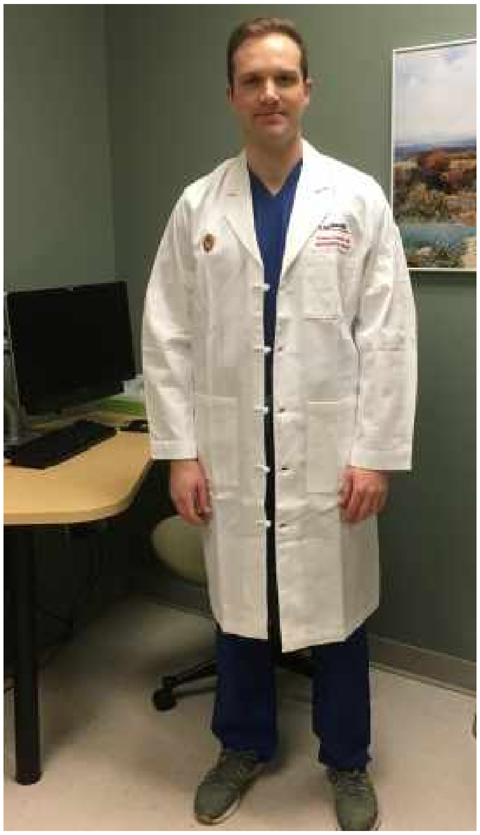

○ ○

Imagine that you are having a clinic visit with the orthopedic surgeon shown below. Which provider seems more likely to **excel in performing the physical parts of surgery**?

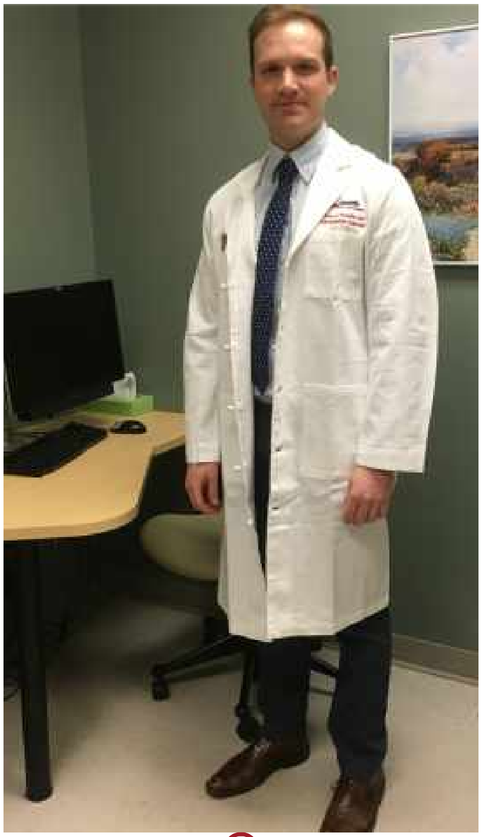

They seem the same

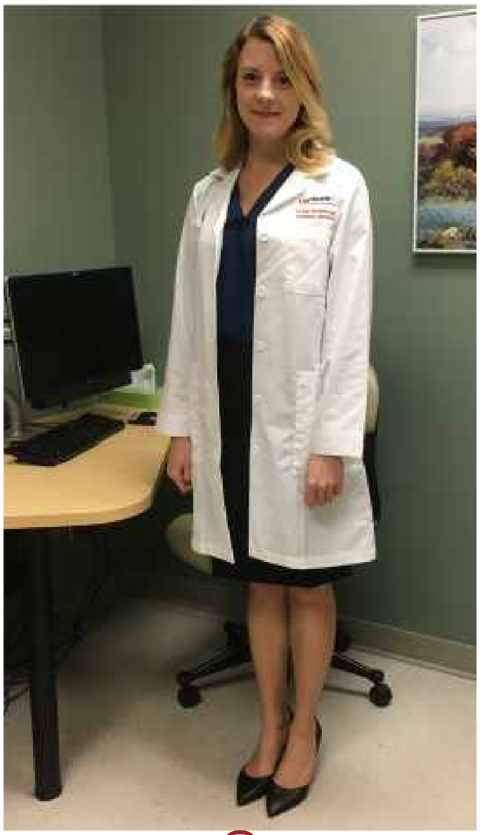

○ ○ ○

Imagine that you are having a clinic visit with the orthopedic surgeon shown below. Which provider seems more likely to **excel in performing the physical parts of surgery**?

They seem the same

○

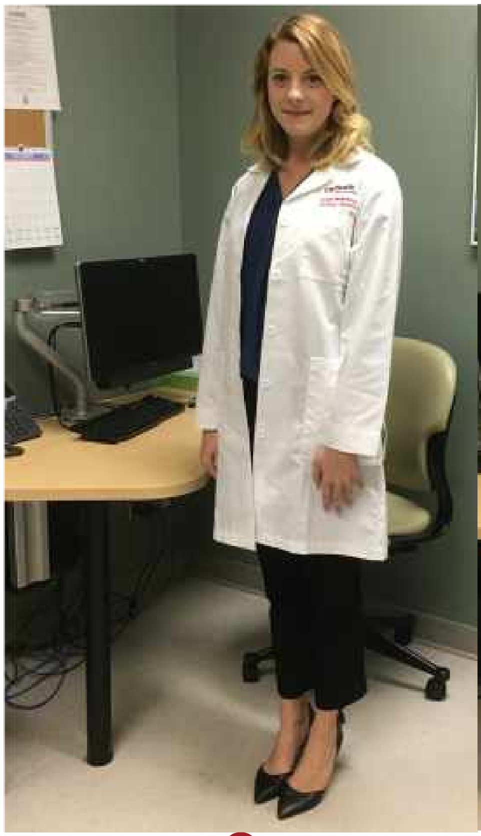

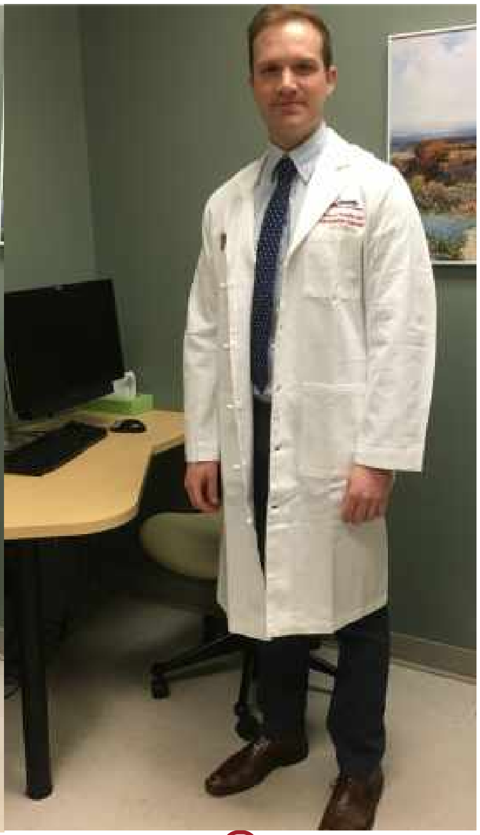

Imagine that you are having a clinic visit with the orthopedic surgeon shown below. Which provider seems more likely to **excel in performing the physical parts of surgery**?

They seem the same

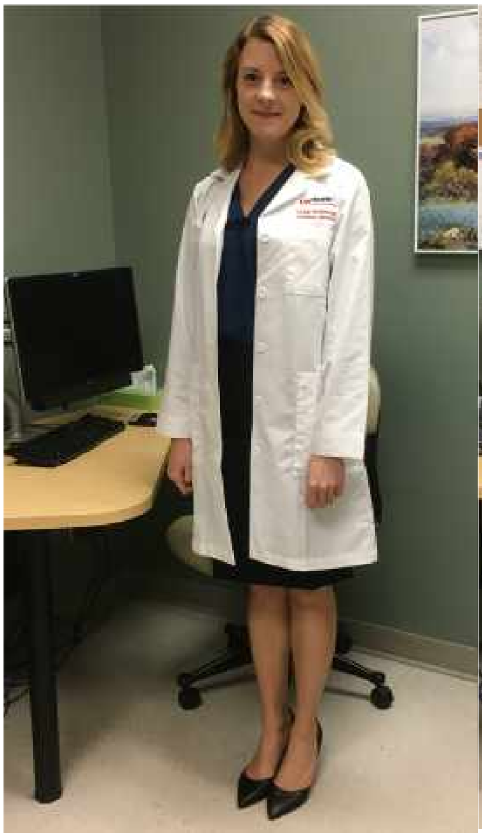

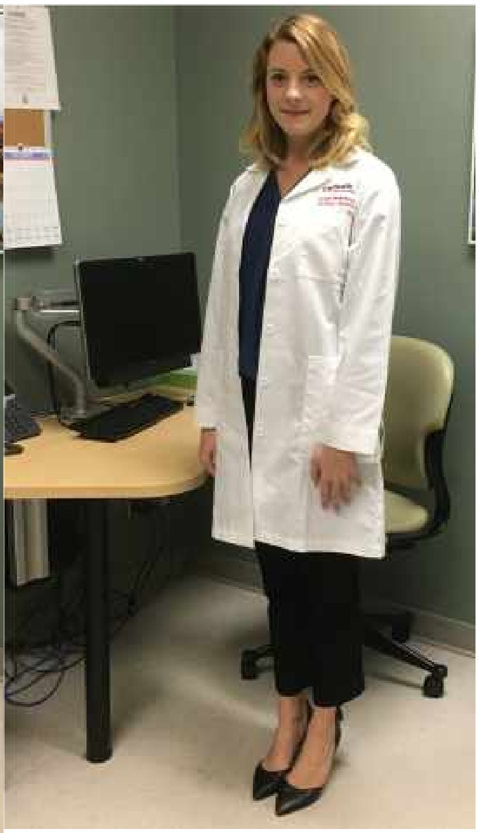

Imagine that you are having a clinic visit with the orthopedic surgeon shown below. Which provider seems more likely to **excel in performing the physical parts of surgery**?

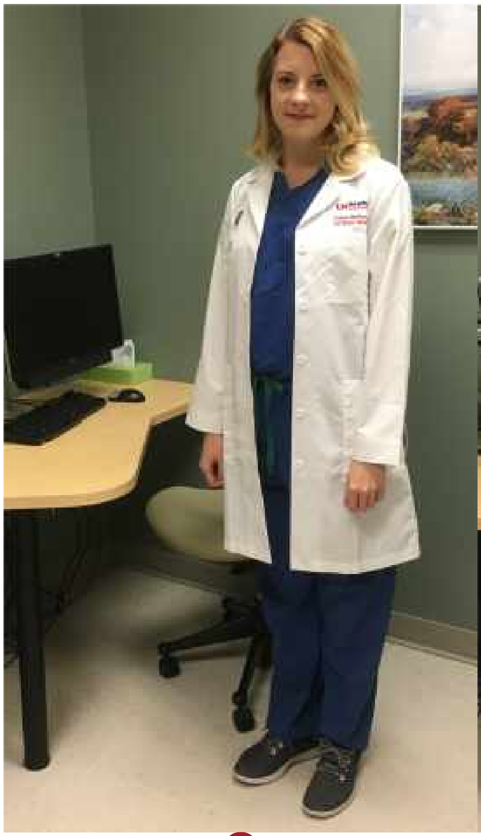

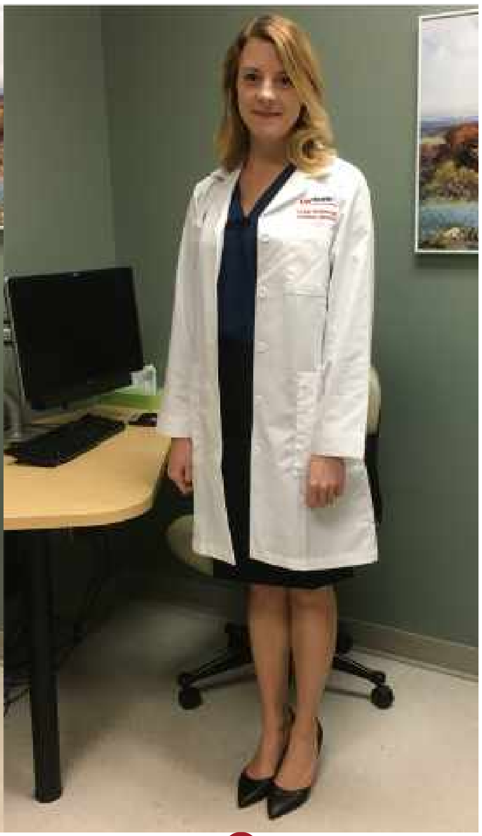

They seem the same

Imagine that you are having a clinic visit with the orthopedic surgeon shown below. Which provider seems more likely to **excel in performing the physical parts of surgery**?

They seem the same

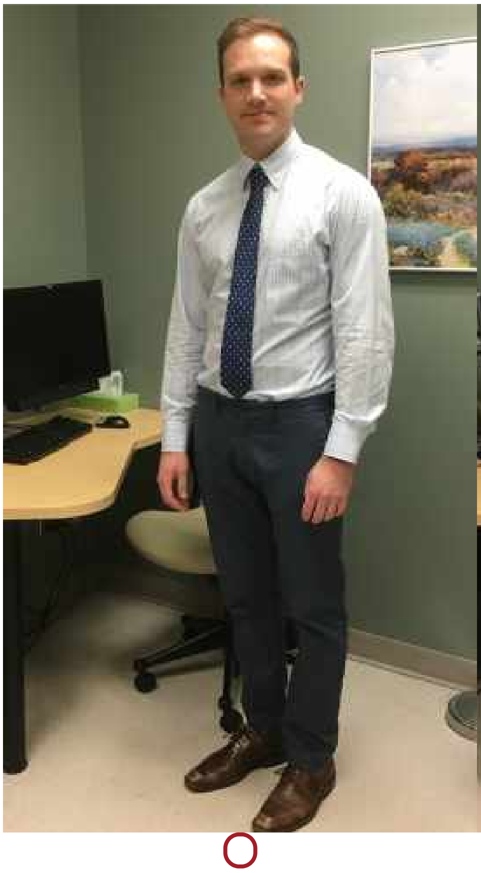

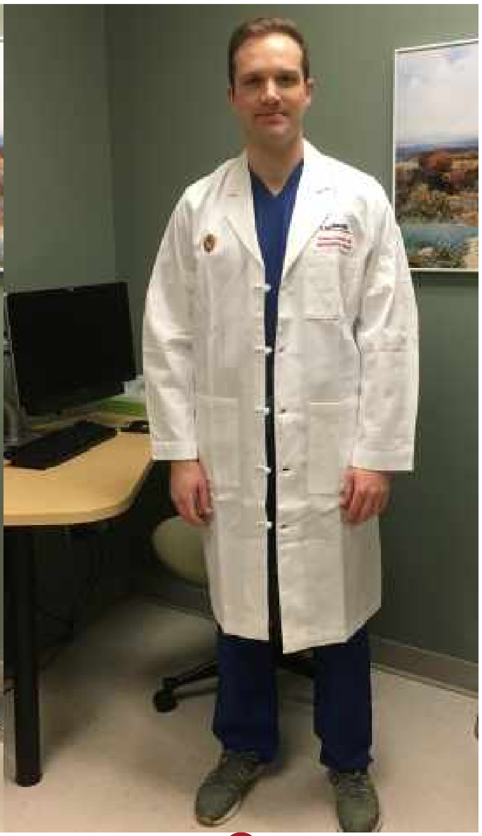

Intro Trustworthy

This fourth section will ask you to pick a surgeon based on who you think is more **trustworthy**.

Trustworthy 1

Imagine that you are having a clinic visit with the orthopedic surgeon shown below. Which provider seems more **trustworthy**?

They seem the same

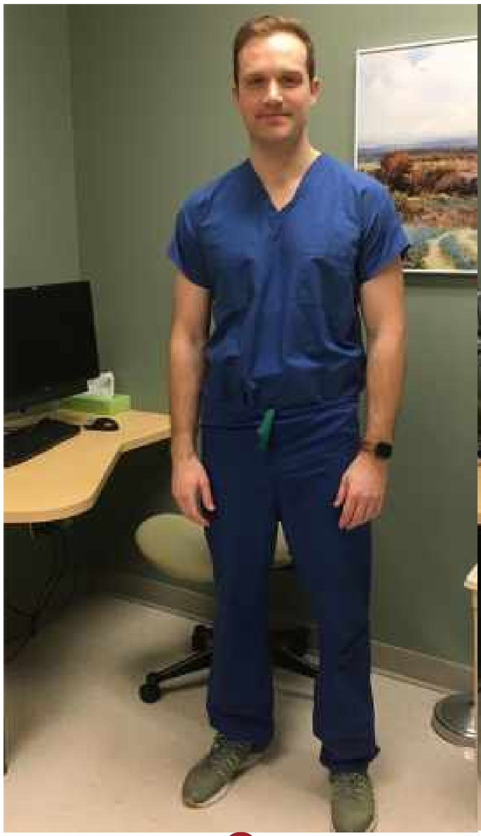

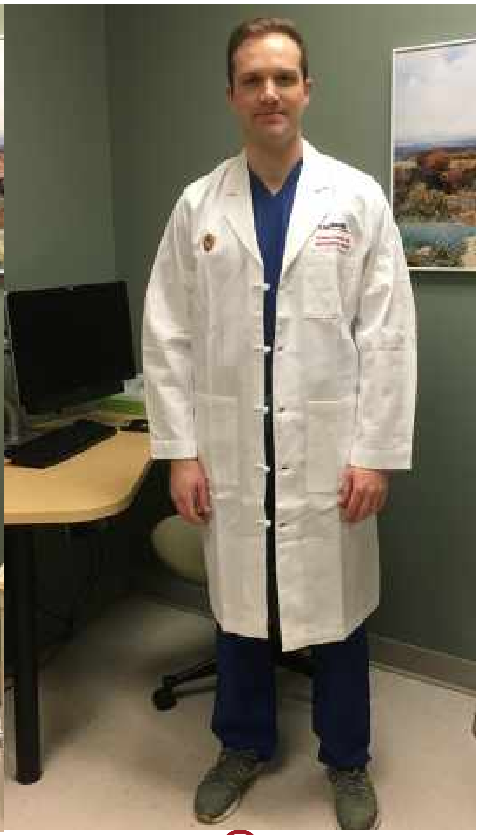

Imagine that you are having a clinic visit with the orthopedic surgeon shown below.
Which provider seems more **trustworthy**?

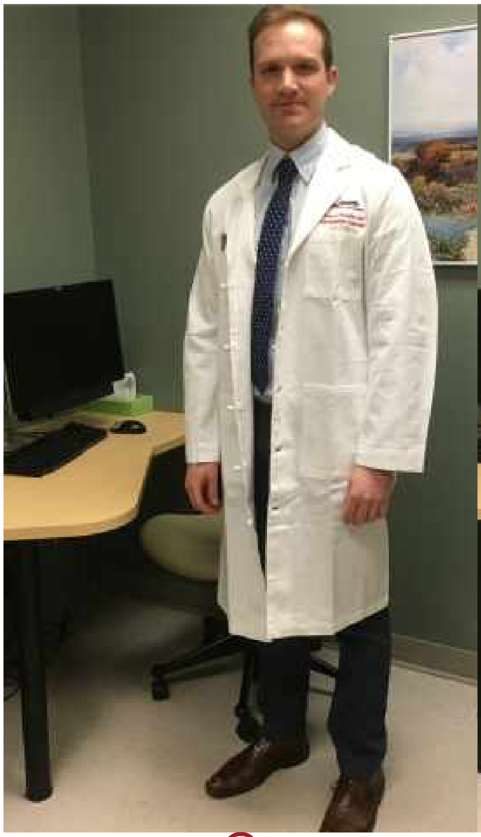

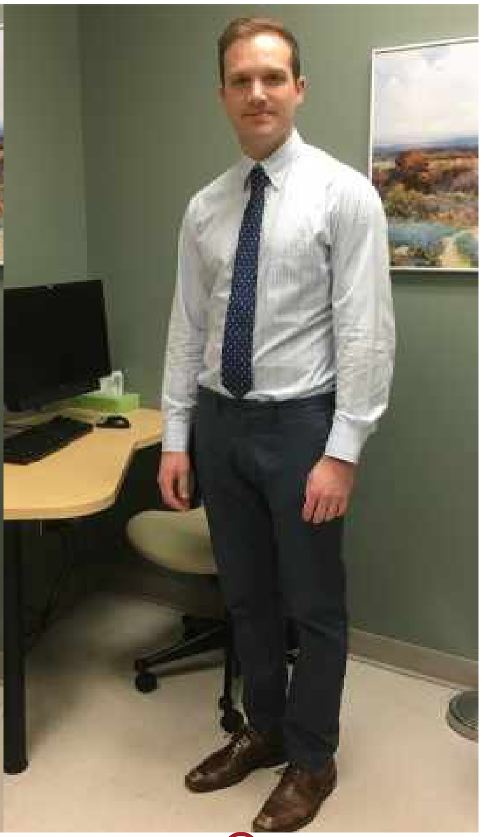

They seem the same

Imagine that you are having a clinic visit with the orthopedic surgeon shown below. Which provider seems more **trustworthy**?

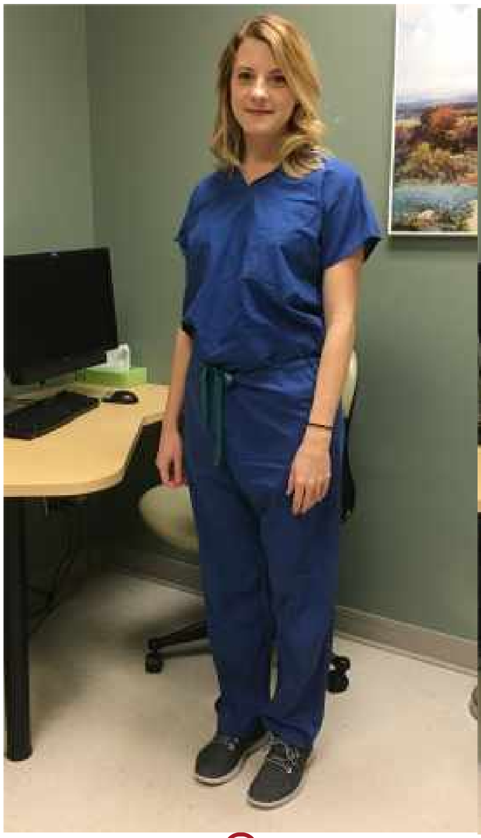

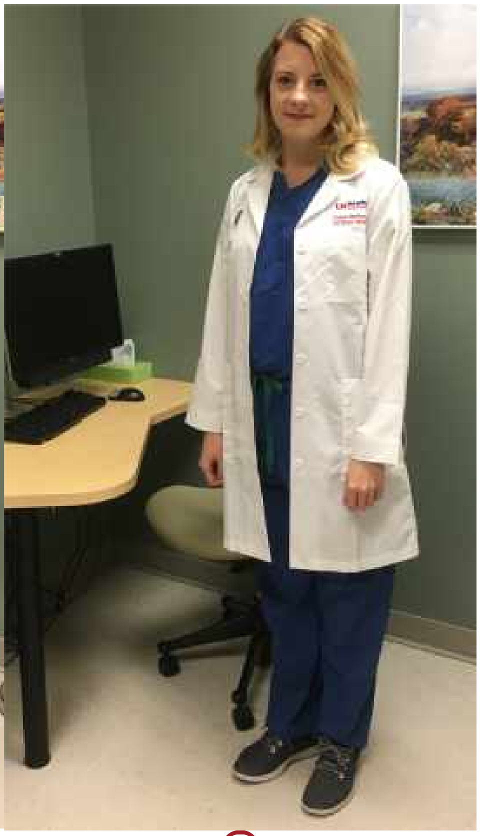

They seem the same

○ ○ ○

Imagine that you are having a clinic visit with the orthopedic surgeon shown below. Which provider seems more **trustworthy**?

They seem the same

○

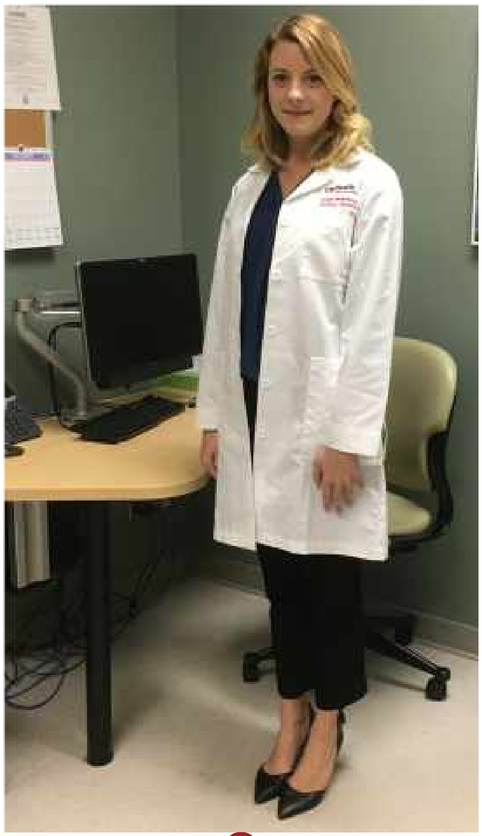

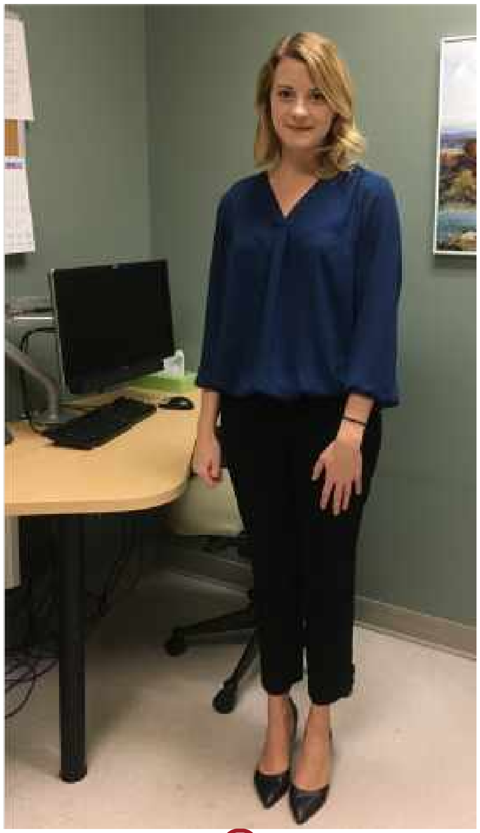

Imagine that you are having a clinic visit with the orthopedic surgeon shown below. Which provider seems more **trustworthy**?

They seem the same

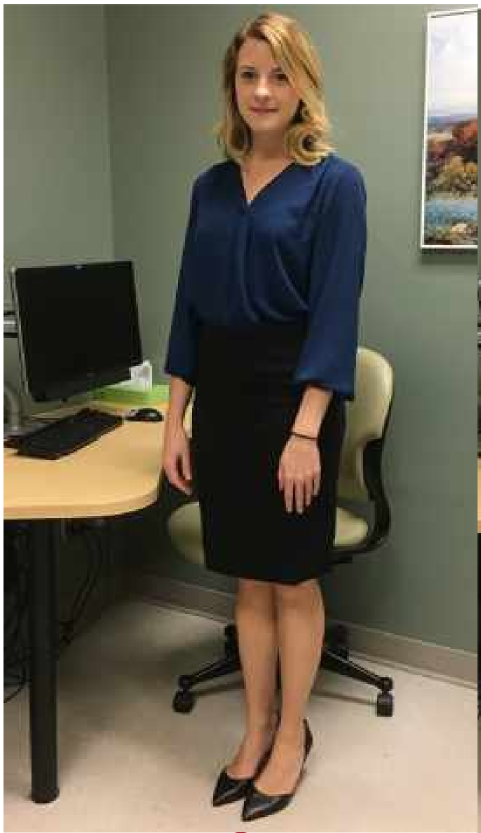

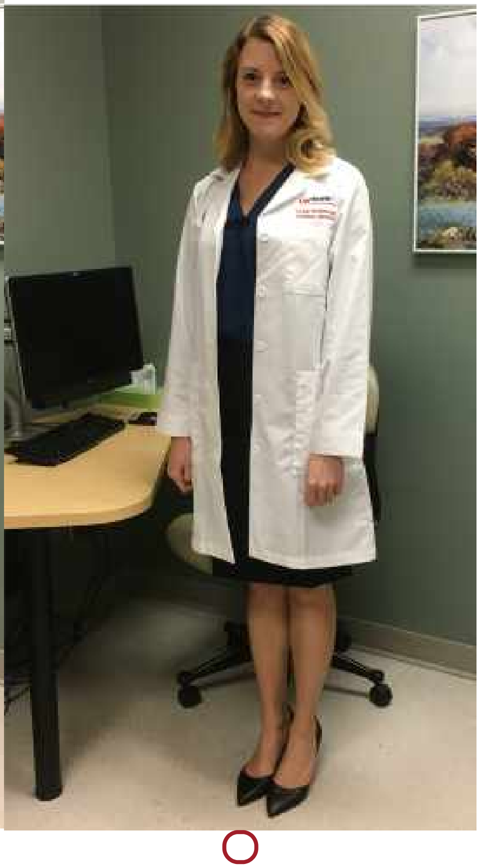

Imagine that you are having a clinic visit with the orthopedic surgeon shown below.
Which provider seems more **trustworthy**?

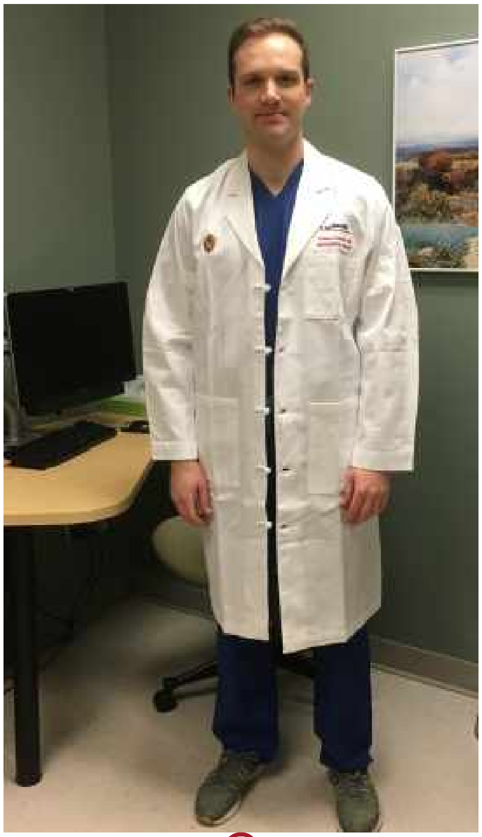

They seem the same

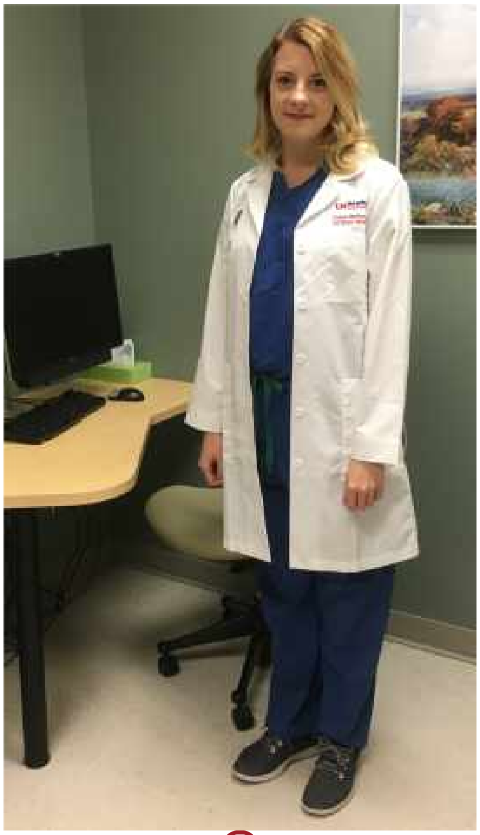

Imagine that you are having a clinic visit with the orthopedic surgeon shown below.
Which provider seems more **trustworthy**?

They seem the same

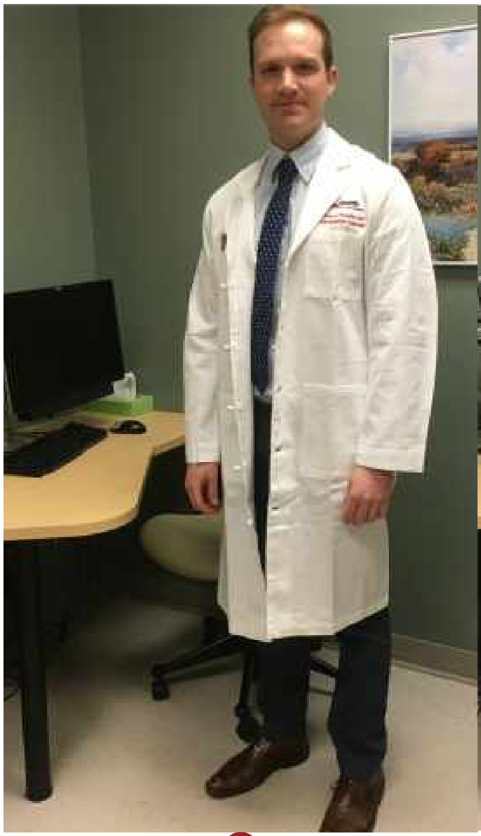
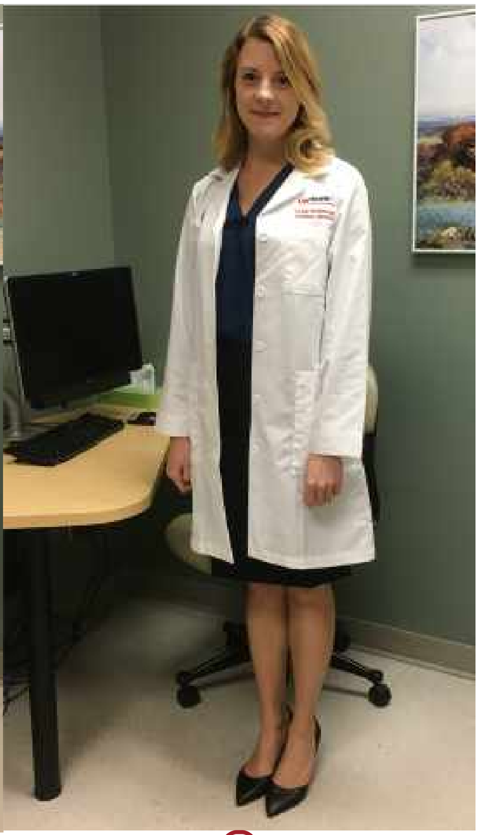

Imagine that you are having a clinic visit with the orthopedic surgeon shown below. Which provider seems more **trustworthy**?

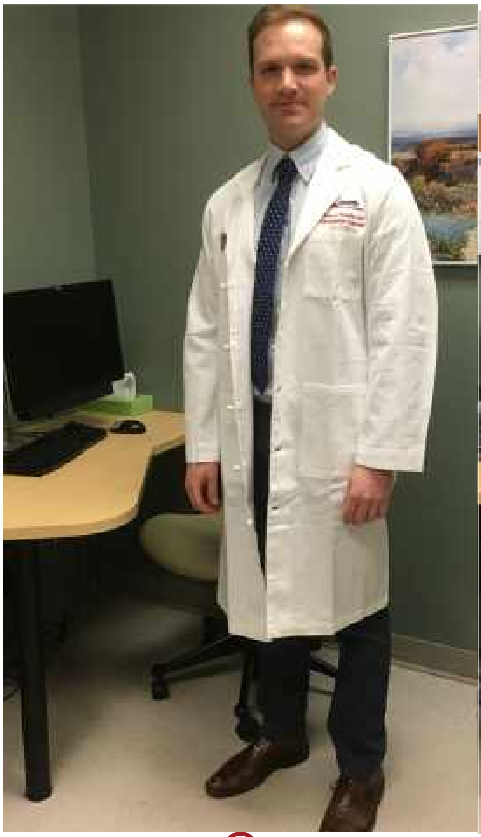
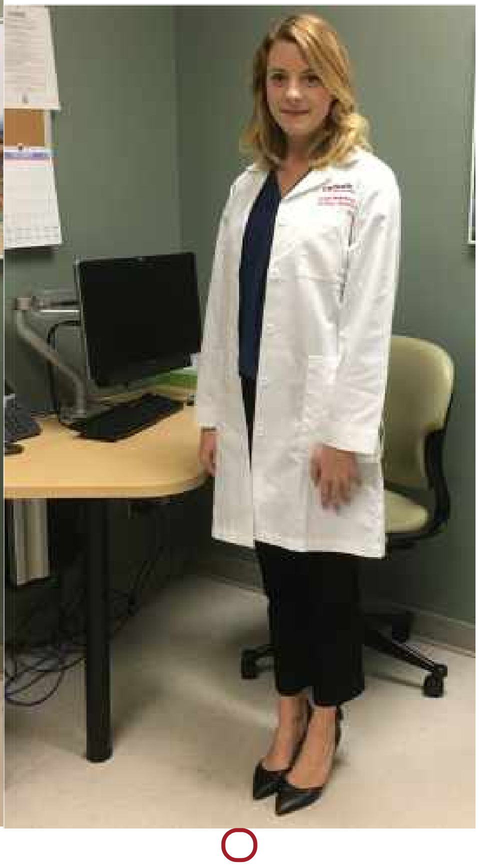

They seem the same

Imagine that you are having a clinic visit with the orthopedic surgeon shown below. Which provider seems more **trustworthy**?

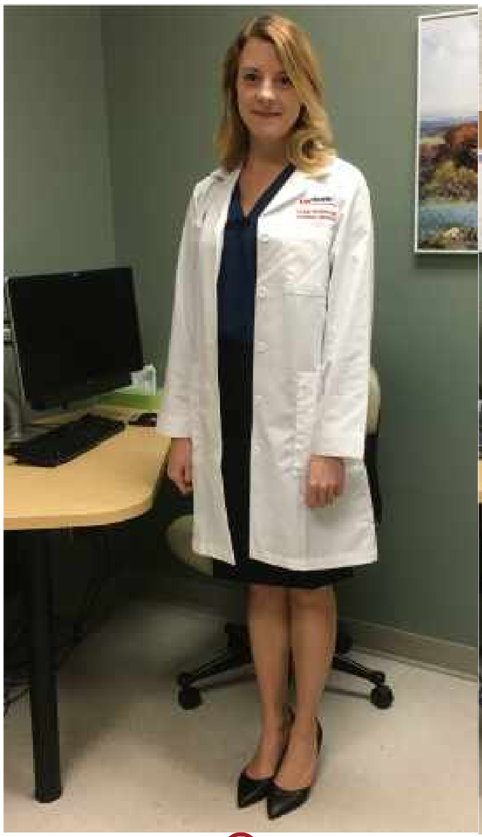

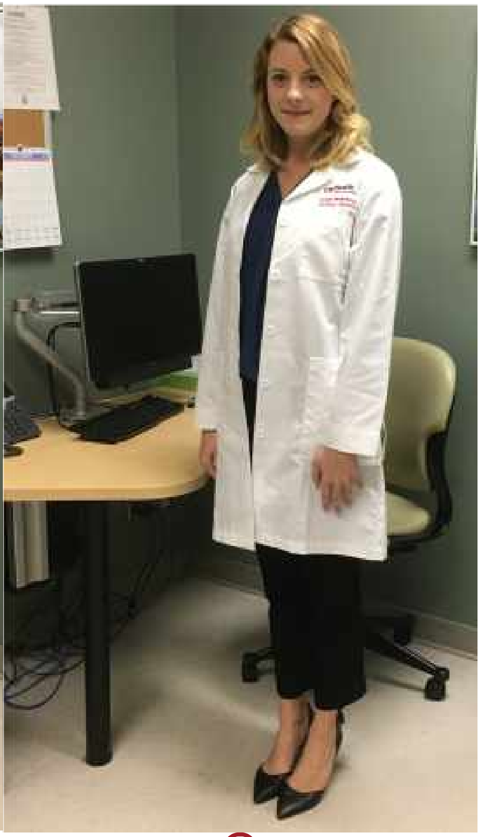

They seem the same

Imagine that you are having a clinic visit with the orthopedic surgeon shown below. Which provider seems more **trustworthy**?

They seem the same

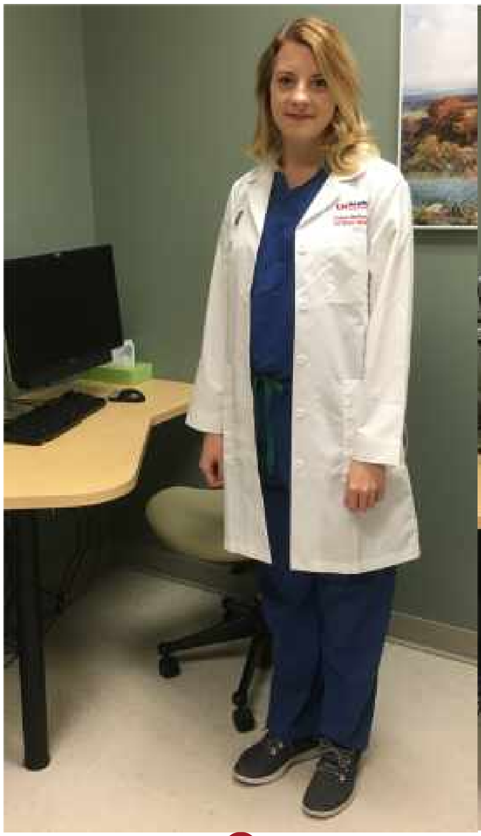

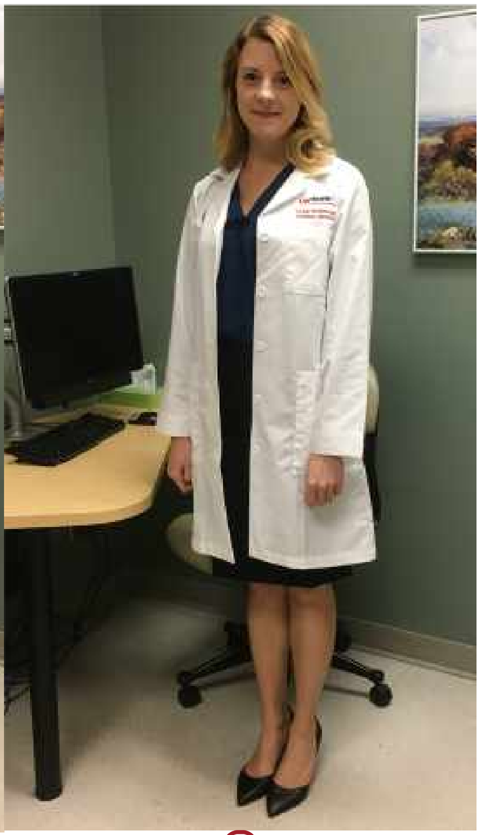

Imagine that you are having a clinic visit with the orthopedic surgeon shown below. Which provider seems more **trustworthy**?

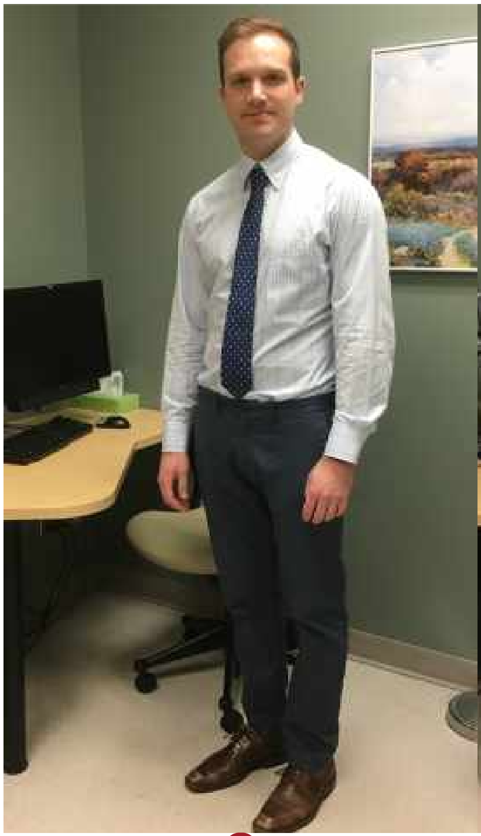

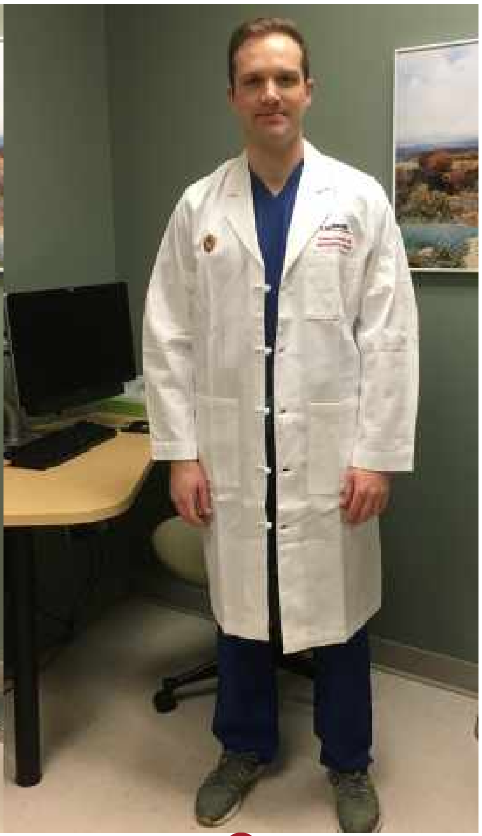

They seem the same

End

Thank you for participating in our survey. There are three final questions.

Think back to your last healthcare experience. Did the providers you met introduce themselves by title or role on the healthcare team?

- Always
- Sometimes
- Rarely
- Never

Is your primary care doctor (PCP, family medicine doctor, or internal medicine doctor):

- Male
- Female
- Do not have a primary care doctor
- Prefer not to say

Do you recognize the models featured in these images?

- No
- Yes

Where do you recognize these models from?

Powered by Qualtrics
